# Areal differences in depth cue integration between monkey and human

**DOI:** 10.1371/journal.pbio.2006405

**Published:** 2019-03-29

**Authors:** Marcelo Armendariz, Hiroshi Ban, Andrew E. Welchman, Wim Vanduffel

**Affiliations:** 1 Laboratory of Neuro- and Psychophysiology, Department of Neurosciences, KU Leuven Medical School, Leuven, Belgium; 2 Center for Information and Neural Networks, National Institute of Information and Communications Technology, Osaka, Japan; 3 Graduate School of Frontier Biosciences, Osaka University, Osaka, Japan; 4 Department of Psychology, University of Cambridge, Cambridge, United Kingdom; 5 Athinoula A. Martinos Center for Biomedical Imaging, Massachusetts General Hospital, Charlestown, Massachusetts, United States of America; 6 Department of Radiology, Harvard Medical School, Boston, Massachusetts, United States of America; 7 Leuven Brain Institute, Leuven, Belgium; Vanderbilt University, United States of America

## Abstract

Electrophysiological evidence suggested primarily the involvement of the middle temporal (MT) area in depth cue integration in macaques, as opposed to human imaging data pinpointing area V3B/kinetic occipital area (V3B/KO). To clarify this conundrum, we decoded monkey functional MRI (fMRI) responses evoked by stimuli signaling near or far depths defined by binocular disparity, relative motion, and their combination, and we compared results with those from an identical experiment previously performed in humans. Responses in macaque area MT are more discriminable when two cues concurrently signal depth, and information provided by one cue is diagnostic of depth indicated by the other. This suggests that monkey area MT computes fusion of disparity and motion depth signals, exactly as shown for human area V3B/KO. Hence, these data reconcile previously reported discrepancies between depth processing in human and monkey by showing the involvement of the dorsal stream in depth cue integration using the same technique, despite the engagement of different regions.

## Author summary

In everyday life, we interact with a three-dimensional world that we perceive via our two-dimensional retinas. Our brain can reconstruct the third dimension from these flat retinal images using multiple sources of visual information, or cues. The horizontal displacement of the two retinal images, known as binocular disparity, and the relative motion between different objects are two important depth cues. However, to make the most of the information provided by each cue, our brains must efficiently integrate across them. To examine this process, we used neuroimaging in monkeys to record brain responses evoked by stimuli signaling depths defined by either binocular disparity or relative motion in isolation, and also when the two cues are combined congruently or incongruently. We found that cortical area MT in monkeys is involved in the fusion of these two particular depth cues, in contrast to previous human imaging data that pinpoint a more posterior cortical area, V3B/KO. Our findings support the existence of depth cue integration mechanisms in primates; however, this fusion appears to be computed in slightly different areas in humans and monkeys.

## Introduction

Visual environments provide a range of cues that allow the brain to extract depth structure from the ambiguous images projected onto the two-dimensional (2D) retinas. A fundamental challenge in visual neuroscience is to understand how this 2D information is processed and integrated, to allow the viewer to perceive and act in a three-dimensional (3D) world. While multiple regions of the macaque [1] and human [2] brain have been found to respond to images that depict depth, it is only recently that we have begun to understand how information from different signals is fused together. While many studies demonstrated that regions of cortex could respond to information conveyed by two different cues (such as depth from binocular disparity, and depth from motion), this alone does not imply that information is fused into a common representation. For instance, information from the two cues might be locally segregated within the cortex. Differentiating responses to fused versus independent signals requires careful assessment of neural responses to presentations of stimuli in which information from the two different cues is manipulated independently.

Electrophysiological recordings from macaque middle temporal (MT) area have suggested representation of surface structures defined by combinations of binocular disparity and relative motion cues [3]. This is consistent with a large number of studies that have highlighted the importance of area MT in signalling structured motion information [4–6] and disparity signals [7–9]. Surprisingly, however, human imaging identified a different neural locus for the fusion of depth signals from disparity and motion [10,11]. Specifically, dorsal visual area V3B/kinetic occipital area (V3B/KO) [12,13] which is located more caudally relative to human MT, showed neuronal responses that match the predictions of cue fusion using multiple tests. What lies behind the apparent discrepancy between the neural locus of fusion in humans and macaques? Is it a difference between species or is it merely technique related: functional MRI (fMRI) versus single unit electrophysiology? It is known that putatively homologous areas in human and macaque (i.e., macaque MT and human MT+) may carry partially different functions [14–16]. It is an open question, however, whether this also holds for cue fusion mechanisms. Here, we aim to rule out differences induced by technique by using comparative brain imaging [5,17,18], thereby exploiting stimuli, experimental designs, and analysis tools previously used in human studies [10] to test for responses in the macaque brain.

**Fig 1:**
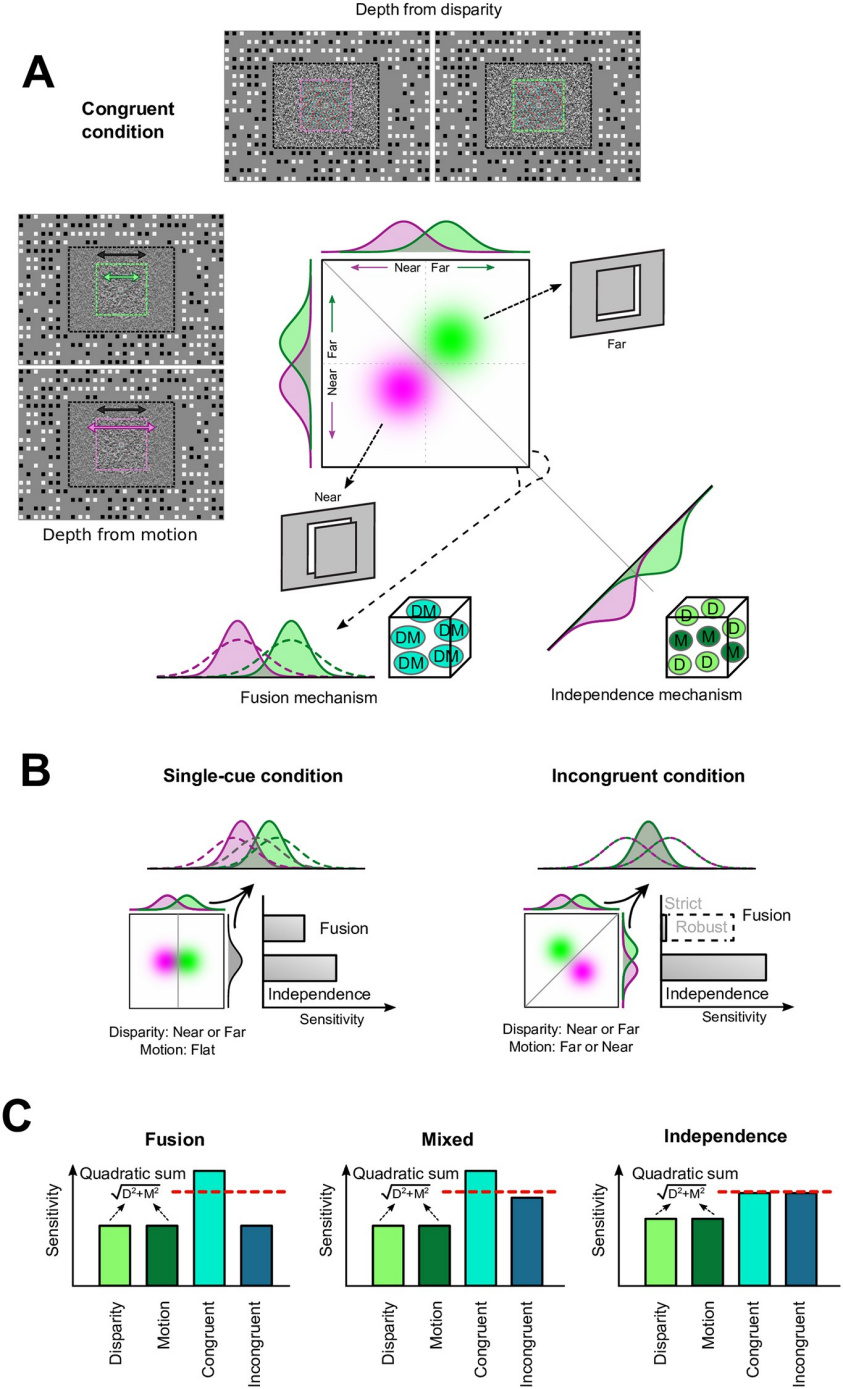
Schematics of stimuli, fusion, and independence mechanisms. (A) The depth of a central target can be defined by disparity and/or motion. Congruent stimuli (disparity and motion presented consistently) are represented as bivariate Gaussian distributions (magenta versus green blobs for near versus far stimuli, respectively). A single cue detector would sense depth along only one dimension (disparity or motion detector): distinguishing the stimuli in this case depends on making a judgment using the marginal distribution (illustrated along the top and left-hand sides of the disparity-motion space). A fusion mechanism (bottom left) combines disparity and motion distributions into a single dimension: this reduces the variance of the combined estimate (solid distributions) relative to the components (dotted distributions). The independence mechanism (bottom right) finds the optimal separating boundary between the stimuli: this increases the separation between the distributions to improve discrimination performance; this corresponds to the quadratic sum of performance along the component axes (by the Pythagorean theorem this means greater separation along the diagonal). Black, magenta, and green dashed lines overlaying the stimuli (not shown during the experiment) are used here to delineate the reference plane and the near and far target planes, respectively. Black, magenta, and green arrows represent the amount of displacement of the reference and target planes. Both dotted planes move sinusoidally (from left to right and vice versa) within the margins determined by the squares of the background (never overlapping with them). (B) Performance of the fusion (left) versus independence (right) mechanisms for the single-cue and incongruent-cue conditions. In both scenarios, the fusion mechanism is compromised and performance decreases, but the independence mechanism is unaffected because depth differences are detected independently. (C) Decoding predictions for an area that responds (ideally) based on fusion or independence. An example of a hypothetical mixed neuronal population response (i.e., neurons tuned to independent cues or to fusion) is shown in the middle panel. Red dotted line depicts the quadratic summation of the marginal cues. D, disparity; M, relative motion; DM, consistent combination of disparity and motion. *Figure was adapted from Welchman*, *2016* [2].

Identifying neural responses for integrated depth signals requires an experimental design that allows us to differentiate collocated, but independent, responses to two different depth cues from an integrated representation that fuses the signals together. To this end, we used random dot displays depicting near or far depth positions of a planar target square relative to its surround. We represent these stimuli as bivariate probability density functions in the space of disparity-motion stimuli (i.e., green and magenta blobs in Fig 1A). By manipulating dot positions in the two eyes (binocular disparity) and differences in the target’s speed relative to its surround (relative motion) we could produce different impressions of depth. Using this stimulus space, we created four conditions in which the target’s near versus far depth was defined by (1) Disparity (where the motion cue indicated zero depth); (2) Motion (where the disparity cue indicated a flat surface in the fronto-parallel plane); (3) Congruent cues (where disparity and motion both indicated the same depth); or (4) Incongruent cues (where disparity indicated one depth position [e.g., near] and motion indicated the other [e.g., far]).

To understand the experimental logic, we outline the way in which fusion versus independence representational schemes should be engaged by these stimuli. First, it is important to understand that when two cues specify the same depth arrangement (Congruent condition), performance is expected to be best under both scenarios, but for different reasons. For an optimal fusion mechanism, information from disparity and motion is averaged together to produce a depth estimate with lower variance (Fig 1A, left). By contrast, an optimal independence mechanism uses the outputs of separate detectors for the two cues. This corresponds to finding the maximal separation between the two stimuli (Fig 1A, right), which can be intuitively computed as the Pythagorean quadratic sum of the separations along the disparity and motion dimensions. Thus, stimuli are more discriminable because their effective separation is increased (Fig 1A, right).

To distinguish fusion from independence, we can measure performance when we manipulate the conflict between the depth information specified by the two cues. First, “single” cue performance (i.e., conditions [1] Disparity or [2] Motion) are useful because they involve one cue indicating no difference in depth between pairs of stimuli (Fig 1B, left). In this case, the independence mechanism effectively ignores the cue signalling zero depth (e.g., geometrically, the hypotenuse can never be shorter than one of the catheti), while the fusion mechanism is compromised because it averages together one signal that specifies depth (e.g., near) with another that indicates a flat surface. Second, incongruent stimuli can cause radically different responses from the two mechanisms. For an independence mechanism, sensitivity should be comparable to that of congruent stimuli: the separation between the two can become greater for incongruent stimuli, for which the cues specify an opposite depth sign (Fig 1B, right). The independence mechanism is not affected by incongruence, as the depth sign is effectively ignored (i.e., the Pythagorean separation still increases whether the cues agree or disagree). However, the fusion mechanism is affected: a strict fusion mechanism could be completely insensitive, although (more realistically) a robust fusion mechanism would revert to the sensitivity of a single cue component.

Using the responses to the different stimuli, we can generate a set of predictions for performance under the fusion and independence scenarios (Fig 1C). For the fusion mechanism, we would expect sensitivity to depth differences in the congruent case to exceed the quadratic summation of performance for the single cue cases. This is because the fusion mechanism is compromised by the conflicts in the single cue stimuli. In addition, performance for incongruent stimuli will be similar to that for single cues (assuming a robust fusion mechanism). By contrast, for the independence mechanism, we would expect congruent cue performance to match the quadratic summation prediction established from the single cue conditions, and no difference in performance for incongruent cues (Fig 1C, right). While a neuronal population might be weighted towards a depth cue integration mechanism, it would be unrealistic to expect a pure fusion-tuned region. In Fig 1C (middle) we show an example of the response of a hypothetical hybrid population response, in which fusion and independent units are collocated.

Here, we test for cortical regions that respond on the basis of cue fusion using functional brain imaging in monkeys and compare that with previous results obtained in humans [10]. We use multivoxel pattern analysis to quantify sensitivity to differences in brain activity evoked by stimuli depicting different depth configurations and contrast empirical performance with the predictions for independence versus fusion.

## Results

To identify areas involved in processing depth cues in the monkey cortex, we started by performing a searchlight analysis that discriminated fMRI responses within an aperture that was moved systematically through the cortex. In particular, we used fMRI monocrystalline iron oxide nanoparticle (MION) responses measured while subjects were presented with stimuli depicting near versus far depths defined on the basis of binocular disparity, relative motion, and their combination. We quantified the discriminability of fMRI responses by training a support vector machine (SVM) to classify patterns of activity evoked by stimuli depicting near versus far depth configurations.

**Fig 2:**
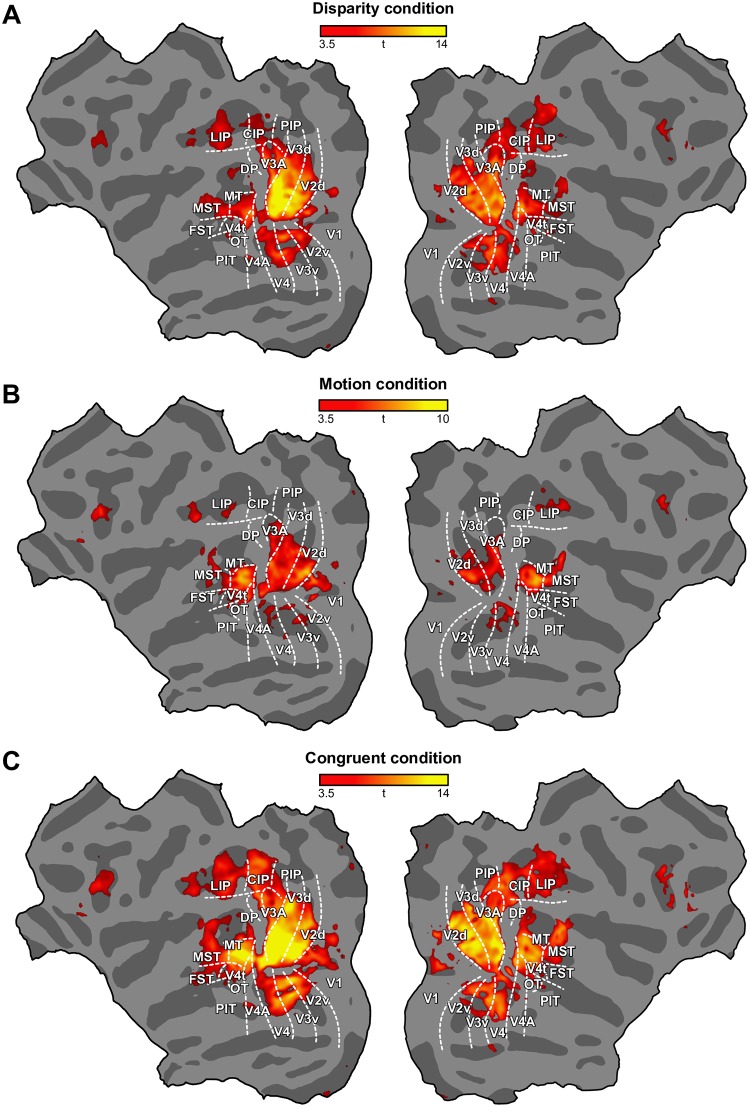
Whole-brain searchlight analyses for disparity, motion, and congruent conditions in monkey. Flat maps showing the left and right monkey cortex. Borders between areas are defined by retinotopic mapping and indicated by the white dotted lines. Sulci/gyri are coded in dark/light gray. Results of a searchlight classifier analysis that moved iteratively throughout the entire volume of cortex, discriminating between near and far depth positions (group data, *N* = 2), are presented. The color code represents the *t* value of the classification accuracies obtained for depths defined by (A) disparity, (B) relative motion, and (C) the congruent combination of disparity and motion. The underlying data for the figures can be found at https://doi.org/10.5061/dryad.6pm117m. CIP, caudal intraparietal area; DP, dorsal prelunate area; FST, fundus of the superior temporal sulcus area; LIP, lateral intraparietal area; MST, medial superior temporal sulcus area; MT, middle temporal area; OT, occipitotemporal area; PIP, posterior intraparietal area; PIT, posterior inferotemporal area; V1, primary visual cortex; V2d, dorsal secondary visual area; V2v, ventral secondary visual area; V3A, visual area 3A; V3d, dorsal visual area 3; V3v, ventral visual areas 3; V4, visual area 4; V4A, visual area 4A; V4t, transitional visual area 4.

We projected the results of this analysis onto a flattened representation of the cortex (Fig 2), thereby producing a searchlight map for near versus far classification for each cue. This suggested that substantial parts of the visual cortex contained fMRI signals that could support reliable stimulus classification. For the motion cue, we found activity that supported reliable classification mostly in dorsal regions of the visual cortex (V2d, V3d, V3A), parietal areas, lateral intraparietal area (LIP) and anterior intraparietal area (AIP), and area MT and its satellites. Relatively weak classification was found in ventral areas of early visual cortex. We also noted, to a lesser extent, meaningful results in frontal areas (frontal eye field [FEF]) and 46. Classification accuracies were higher overall for the disparity cue and, in addition to the dorsal visual areas (unlike motion, also including the dorsal prelunate area [DP]), we observed ample significant classification in ventral areas (V2v, V3v, V4) and the parietal cortex (caudal intraparietal area [CIP], LIP, posterior intraparietal area [PIP]). Classification accuracies were higher still for the combined cue stimulus (disparity and motion). Searchlight maps were consistent across subjects (S1A–S1C and S2A–S2C Figs). Additionally, we computed a searchlight map for the incongruent stimuli in which reliable classification covered similar areas as the congruent condition (S3 Fig). A voxel-versus-voxel comparison of the congruent and incongruent maps was not sensitive enough to observe significant differences.

**Fig 3:**
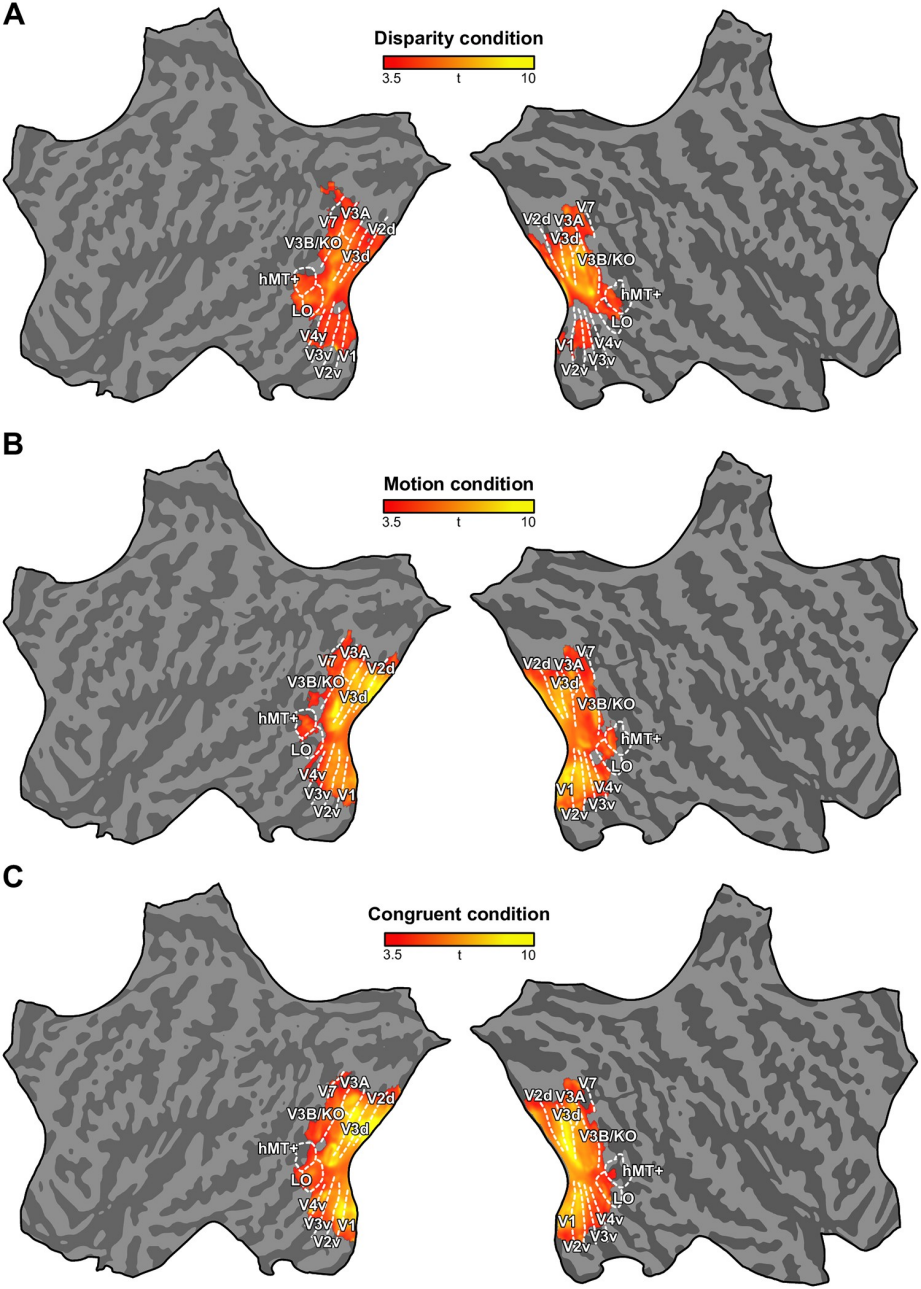
Whole-brain searchlight analyses for disparity, motion, and congruent conditions in human. Flat maps showing the left and right human cortex. Data are from Ban and colleagues, 2012 [10]. Same conventions as in Fig 2. The color code represents the *t* value of the classification accuracies obtained for depths defined by (A) disparity, (B) relative motion, and (C) the congruent combination of disparity and motion (group data, *N* = 20). The map for the incongruent condition is shown in S4 Fig. The underlying data for the figures can be found at https://doi.org/10.5061/dryad.6pm117m. hMT+, human middle temporal area; LO, lateral occipital area; V3B/KO, area V3B, kinetic occipital area.

Similar to monkeys, a wide range of areas in human visual cortex were involved in processing stimuli depicting depth (Fig 3). Human dorsal areas V3d and V3B/KO consistently showed highest reliable decoding across the three conditions. Primary and secondary visual cortex (V1, V2) and ventral areas (V3v, V4) also supported significant classification accuracies, yet were particularly strong when motion-defined depth was presented. In addition, weaker although significant discriminability was also found in dorsal areas V3A and V7. However, whereas our monkey data pointed to MT as an important cortical locus for depth processing, the presumed conglomerate of homologous areas in humans, the MT+ complex [19], showed only partial and fragmentary significant classification across conditions and hemispheres. Similar fragmented response patterns were found in the lateral occipital (LO) cortex. Moreover, in contrast to monkeys, humans exhibited more activity for motion than for disparity. Surprisingly, whereas disparity compared to motion-based classification was qualitatively more pronounced in ventral visual areas in monkeys, the opposite was found in humans.

### Region of interest–based multivoxel patterns analysis

To better assess depth processing in the monkey brain, we adapted methods from a previous human study [10] and confined the fMRI responses to independently defined regions of interest (ROIs; see Materials and methods for definitions). We then trained a machine learning classifier (SVM) to distinguish between near and far voxel activation patterns for each depth cue and across ROIs. For each area, we calculated the performance of the classifier in decoding depth from an independent data set using a leave-one-out cross-validation approach.

**Fig 4:**
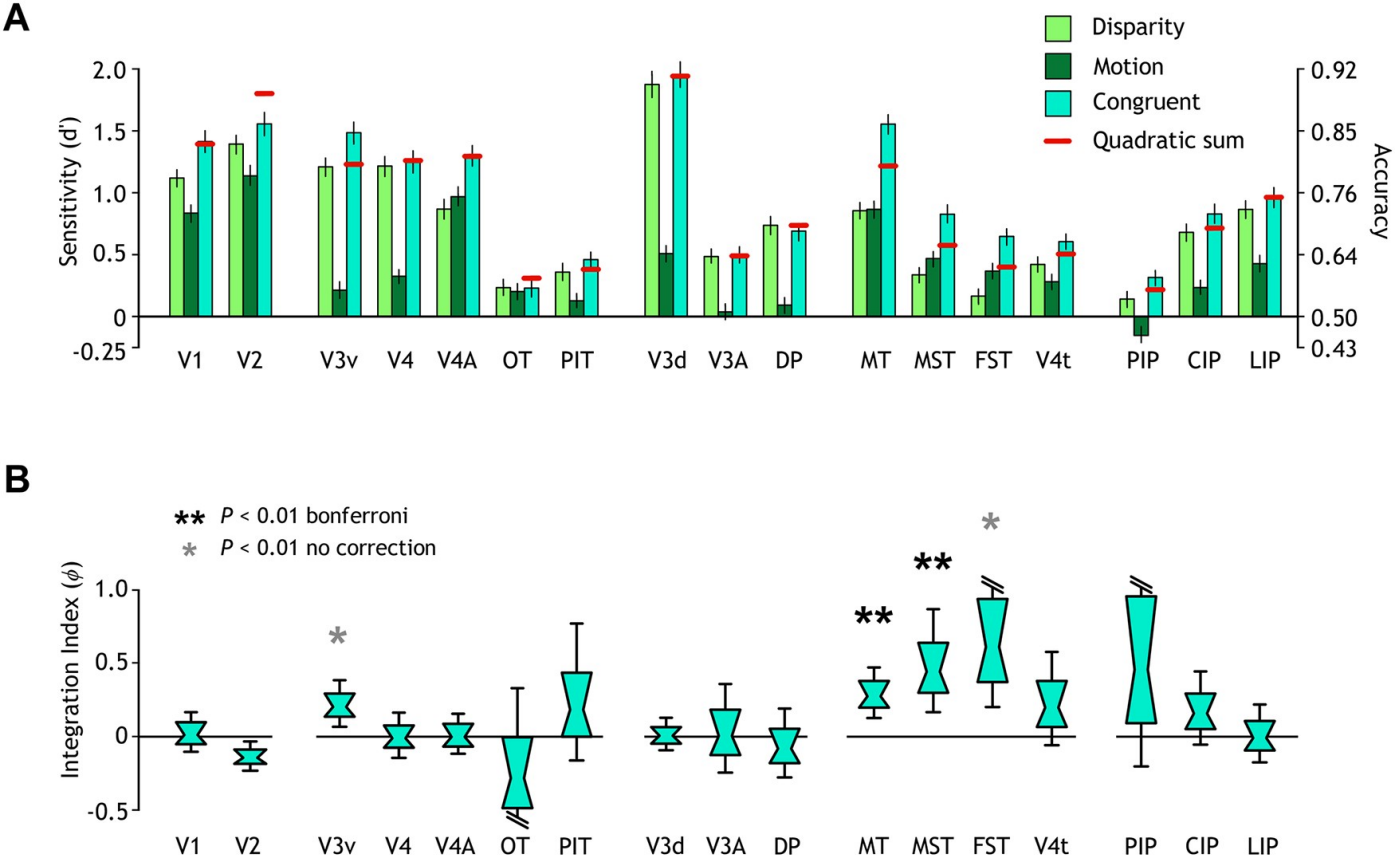
Classification performances and quadratic summation test. (A) Prediction performance (accuracy and sensitivity) for near versus far discrimination in different ROIs and for different conditions. The red lines illustrate performance expected from the quadratic summation of prediction sensitivities for the marginal cues. Error bars, SEM. (B) Results as an integration index. A value of zero indicates the minimum bound for fusion (the prediction based on quadratic summation). Data are presented as notched distribution plots. The center of the “bowtie” represents the median, the greenish area depicts 68% confidence values, and the upper and lower error bars 95% confidence intervals. The underlying data for the figures can be found at https://doi.org/10.5061/dryad.6pm117m. CIP, caudal intraparietal area; DP, dorsal prelunate area; FST, fundus of the superior temporal sulcus area; LIP, lateral intraparietal area; MST, medial superior temporal sulcus area; MT, middle temporal area; OT, occipitotemporal area; PIP, posterior intraparietal area; PIT, posterior inferotemporal area; ROI, region of interest.

Consistent with the searchlight analyses described above, depth defined by congruent stimuli was reliably decoded in most of the ROIs (Fig 4A), but performance varied across areas. This widespread sensitivity to different cues throughout visual cortex does not indicate explicit encoding of depth. The SVM may decode low-level image features, rather than depth per se. Discrimination performance was higher across the three conditions in early visual areas (V1, V2), ventral area V4A, dorsal V3, and MT (and its satellites). Although most of the areas showed lower sensitivity for both single cues compared with the concurrent stimulus, motion classification was particularly poor (<56%) in the more dorsal regions (V3A, DP, PIP, and CIP).

### fMRI quadratic summation

Our main interest was not to compare classification accuracies of single and combined cue conditions across regions (because these are influenced by a range of factors besides neural activity, such as our ability to measure fMRI activity with the same sensitivity in different anatomical locations—which basically holds for all fMRI studies comparing signals across areas) but to evaluate the relative performance within each area of the condition in which both disparity and motion concurrently signalled depth.

We compared prediction accuracies for the congruent stimulus relative to a minimum bound for fusion prediction based on the quadratic summation of decoding accuracies for the single cues (see Figs 1 and 4A). We found that fMRI responses were higher than quadratic summation (*P* < 0.01) in areas of the MT cluster (fundus superior temporal [FST], medial superior temporal [MST], and MT) and ventral V3. We quantified the extent of integration across areas using a bootstrapped index (Materials and methods, Eq 1). Values close to zero correspond to the performance expected if information from disparity and motion are collocated but processed independently. A higher value would indicate that a fusion mechanism may be present (see Fig 1C). We found that the integration index in mid-level areas MST and MT significantly exceeded zero (Bonferroni correction for multiple comparisons, *P* < 0.01) (Fig 4B). In addition, we found integration indices above zero (uncorrected threshold) in areas FST and V3v (Table 1) (for human results, see S5 Fig and Ban and colleagues, 2012). Our findings in the MT complex are consistent with decoding improvements for the congruent condition as a result of the fusion of disparity and motion cues (see Fig 1)—although this test alone cannot rule out independences.

**Table 1:** Significance tests for the integration index, congruency, and transfer index.

Area	*P* value		
	Integration index above zero	Congruent versus incongruent	Transfer index from chance
V1	0.4166	0.1323	0.0014
V2	0.9957	0.5755	0.0034
V3v	0.0027	0.0119	0.0901
V4	0.5305	0.9448	0.7851
V4A	0.4949	0.3559	0.0177
OT	0.8483	0.9244	0.9750
PIT	0.1708	0.0066	0.9999
V3d	0.4482	0.1485	**0.0001**
V3A	0.4701	0.9824	0.8874
DP	0.7433	0.9786	0.9993
MT	**0.0001**	**0.0001**	**0.0001**
MST	**0.0002**	0.1077	0.7006
FST	0.0011	0.0213	0.9999
V4t	0.0734	0.9758	0.1672
PIP	0.1053	0.6139	0.9999
CIP	0.0867	0.8908	0.3893
LIP	0.5229	0.6474	0.3355

Probabilities associated with obtaining (i) a value of zero for the fMRI integration index, (ii) zero difference between classification performances for congruent and incongruent conditions, and (iii) no difference between the value of the transfer index compared with random performance. These *P* values are calculated using bootstrapped resampling with 10,000 samples. Bold indicates Bonferroni-corrected significance (*P* < 0.01). MT is the only area passing all tests at Bonferroni-corrected level.

Abbreviations: CIP, caudal intraparietal area; DP, dorsal prelunate area; fMRI, functional MRI; FST, fundus of the superior temporal sulcus area; LIP, lateral intraparietal area; MST, medial superior temporal sulcus area; MT, middle temporal area; OT, occipitotemporal area; PIP, posterior intraparietal area; PIT, posterior inferotemporal area.

### Congruent versus incongruent cues

We added the incongruent condition to perform a second test to assess cue integration mechanisms. Both cues, motion and disparity, were presented simultaneously, but, in this case, depicting opposite depths (one cue signalled “near” and the other “far”). If the representation of depth defined by the two stimulus dimensions (disparity and motion) is independent, this conflicting situation should have no effect on classification performance. Thus, the performance of a machine learning classifier in distinguishing between voxel patterns should be similar for both congruent and incongruent conditions and comparable to the quadratic sum of the component cues. In contrast to this expectation, if fusion is present, the discrimination performance should be lower when motion and disparity conflict. Under strict fusion, performance would diminish below that of either component cue, whereas in robust fusion, performance would revert to the level of one of the two components.

**Fig 5:**
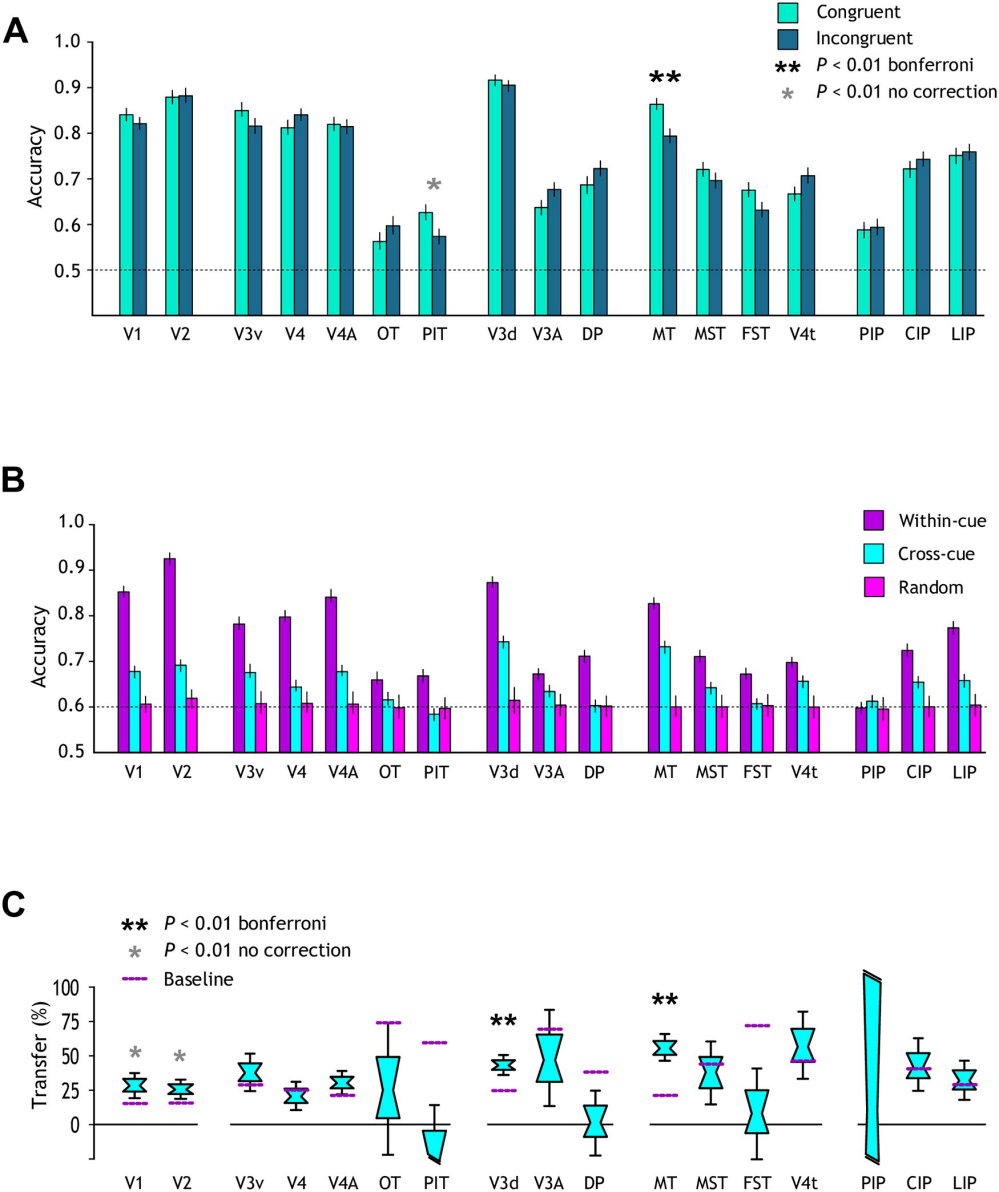
Congruency and transfer test. (A) Prediction accuracy for near versus far classification when cues are congruent or incongruent in different ROIs. The horizontal line at 0.5 corresponds to chance performance. Error bars, SEM; *P < 0.01 uncorrected; **P < 0.01 Bonferroni corrected. (B) Prediction accuracy for the cross-cue transfer analysis in different regions. Classification performances are shown when data were trained and tested with the same cue (within-cue, dark purple), trained with one cue and tested with the other (cross-cue, cyan), and for randomly permuted data (light purple). Error bars, SEM. (C) Data shown as a transfer index. A value of 100% would indicate that prediction accuracies were equivalent for within- and between-cue testing. Distribution plots show the median; cyan area and error bars represent the 68% and 95% confidence intervals, respectively. Purple dotted horizontal lines depict a bootstrapped chance baseline based on the upper 95th percentile for transfer obtained with randomly permuted data. *P < 0.01 uncorrected; **P < 0.01 Bonferroni corrected. The underlying data for the figures can be found at https://doi.org/10.5061/dryad.6pm117m. CIP, caudal intraparietal area; DP, dorsal prelunate area; FST, fundus of the superior temporal sulcus area; LIP, lateral intraparietal area; MST, medial superior temporal sulcus area; MT, middle temporal area; OT, occipitotemporal area; PIP, posterior intraparietal area; PIT, posterior inferotemporal area; ROI, region of interest.

Area MT exhibited a significant drop in performance for the incongruent condition in comparison to the congruent stimulus (Fig 5A). However, classification accuracy was still slightly higher than for single cue conditions. The visual posterior inferotemporal area (PIT) also showed lower prediction accuracy for the incongruent stimulus, although this was not significant after Bonferroni correction. These findings suggest that MT may house a mixed population that contains both units tuned to independent and to fused cues. This may help support a robust fusion process that prevents viewers from becoming completely insensitive to conflicting cues.

### Transfer test

To assess for brain areas involved in a generalized depth representation, we performed a cross-cue transfer test. In particular, we evaluated whether activity patterns evoked by one depth cue provide information about the other. We trained a machine learning classifier to discriminate depth configurations using one cue (for example, disparity), and tested classifier’s prediction using depth responses elicited by the other cue (for example, motion).

For each ROI, we compared the average performance of the classifier in predicting the near versus far stimulus configuration within cues (trained and tested with the same cue) and across cues (trained with one cue and tested with the other) (Fig 5B). To quantify the transfer of information between cues we calculated a bootstrapped index (Materials and methods, Eq 2). A value of one would indicate that prediction accuracy is equal for both within- and between-cue classification, meaning that there is 100% transfer of information. To provide a baseline for the transfer that arises by chance, we conducted the transfer test on randomly permuted data. We calculated random transfer performance 1,000 times for each ROI, and we chose the 95th percentile of the resulting distribution of transfer indices as the significance threshold.

We found transfer indices above the baseline in areas V1, V2, V3d, and MT, although only V3d and MT exceeded the threshold significantly (Bonferroni corrected, *P* < 0.01) (Table 1). Transfer performance of MT was around 55% of that obtained when trained and tested on the same cue (Fig 5C). These results suggest that area MT may play a key role in a more generic representation of depth in the monkey. The same test in humans highlighted dorsal areas V3d and V3B/KO, but did not yield significant results in hMT+ (S6 Fig).

### Composition of the neuronal population explaining cue-fusion results

**Fig 6:**
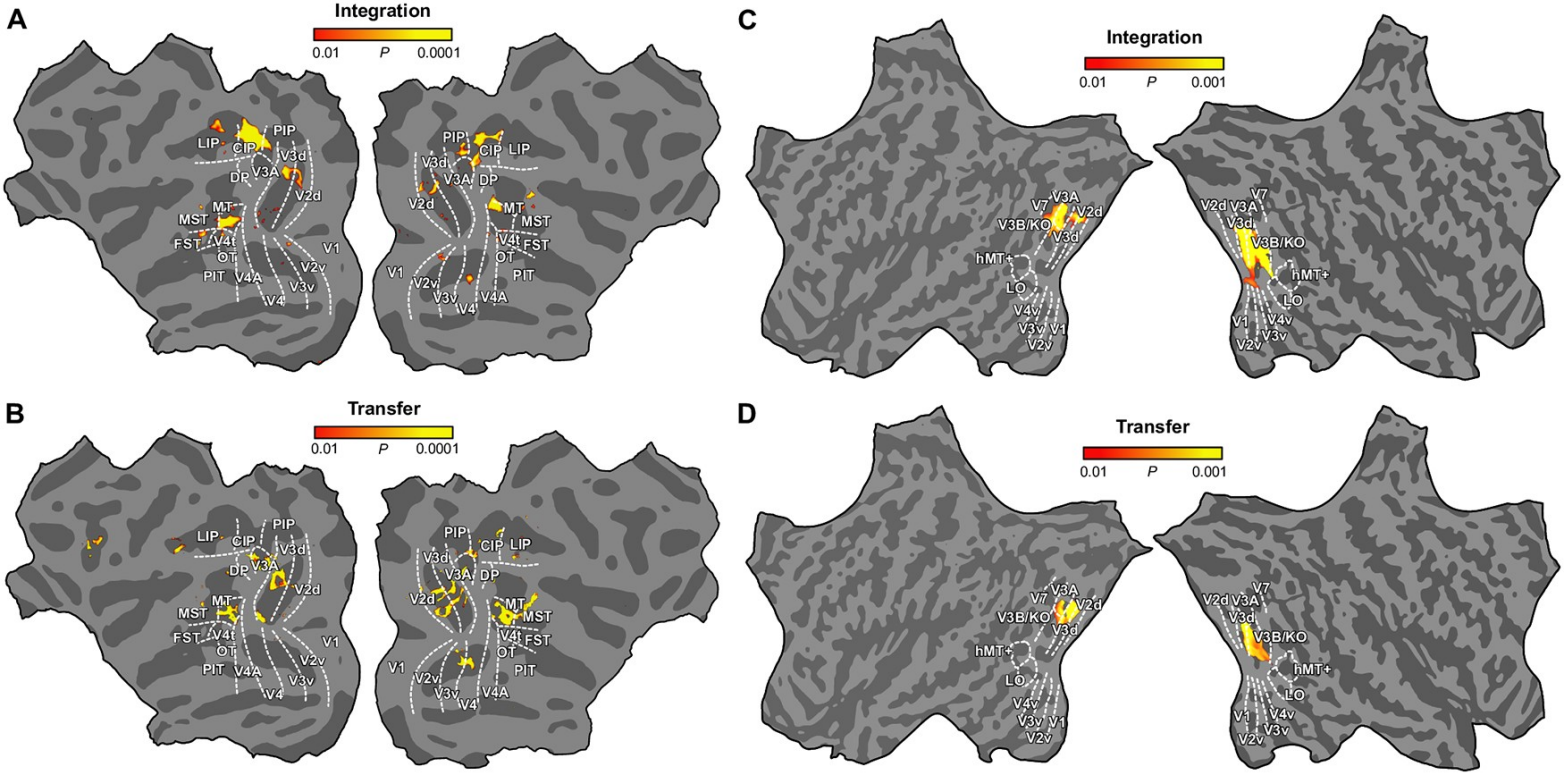
Flat maps for integration and transfer tests based on the searchlight analyses. Integration and transfer test maps for monkeys (A, B) and humans (C, D), calculated from the results of group searchlight classifier analyses. Color code represents the *P* values obtained from the bootstrap distribution of the integration and transfer indices in monkey and human. The underlying data for the figures can be found at https://doi.org/10.5061/dryad.6pm117m. CIP, caudal intraparietal area; DP, dorsal prelunate area; FST, fundus of the superior temporal sulcus area; LIP, lateral intraparietal area; MST, medial superior temporal sulcus area; MT, middle temporal area; OT, occipitotemporal area; PIP, posterior intraparietal area; PIT, posterior inferotemporal area.

So far, we considered scenarios in which neuronal population responses relate either to fusion or independence mechanisms. However, even in areas where we found evidence for depth cue integration (monkey MT and human V3B/KO), it is unlikely that we sampled voxels that respond exclusively to fused signals. To evaluate how different population mixtures might affect decoding results, we used simulations in which we systematically varied the composition of the neuronal population and compared the simulation results with the empirical data (S7 Fig), exactly as in Ban and colleagues, 2012 (see their Fig 6 and Methods section). Whereas in humans the estimated number of fusion units ranges between 50% and 70% in V3B/KO, our simulations suggested that approximately 35% of the neural population in monkey MT might be tuned to fusion. This difference in neuronal composition across species might explain the relative differences in classification performance between human V3B/KO (Fig 6A of Ban and colleagues, 2012) and monkey MT (S7B Fig). In particular, whereas classification performance for the incongruent condition is comparable to that of the single cues in human V3B/KO, the lower percentage of units that contribute to fusion in monkey MT might have caused higher performance for the incongruent condition compared with single cues. Despite these differences, the crucial point is whether the sensitivity for the incongruent condition exceeds the quadratic summation of the single cues. Our statistical tests showed that sensitivity for the incongruent condition was not significantly greater than the quadratic summation in monkey MT nor in human V3B/KO (S10 Fig). In sum, our analyses showed that the ratio of fusion-tuned units in monkey MT is sufficiently high to observe significant differences between the classification performances for congruent and incongruent conditions.

### Searchlight MVPA: Integration and transfer tests

In addition to performing our ROI analysis, we used the searchlight multivoxel pattern analysis (MVPA) approach to confirm that we did not miss important areas involved in cue integration in monkeys (Fig 6A). We first used the integration index and confirmed our ROI-based analysis of area MT as well as revealing responses in CIP and a confined region in the dorsal part of V2 and V3. Searchlight analysis of the human brain (Fig 6C) pointed to area V3B/KO as the only consistent locus for cue integration. Second, we used a searchlight analysis of the cross-cue transfer test. We found that areas V3d and MT supported significant classification of near-far patterns across cues (Fig 6B). In addition to these two areas, V3A also exhibited high transfer indices. For humans, the highest transfer indices were found around the dorsal areas V3d, V3A, and V3B (Fig 6D).

**Fig 7:**
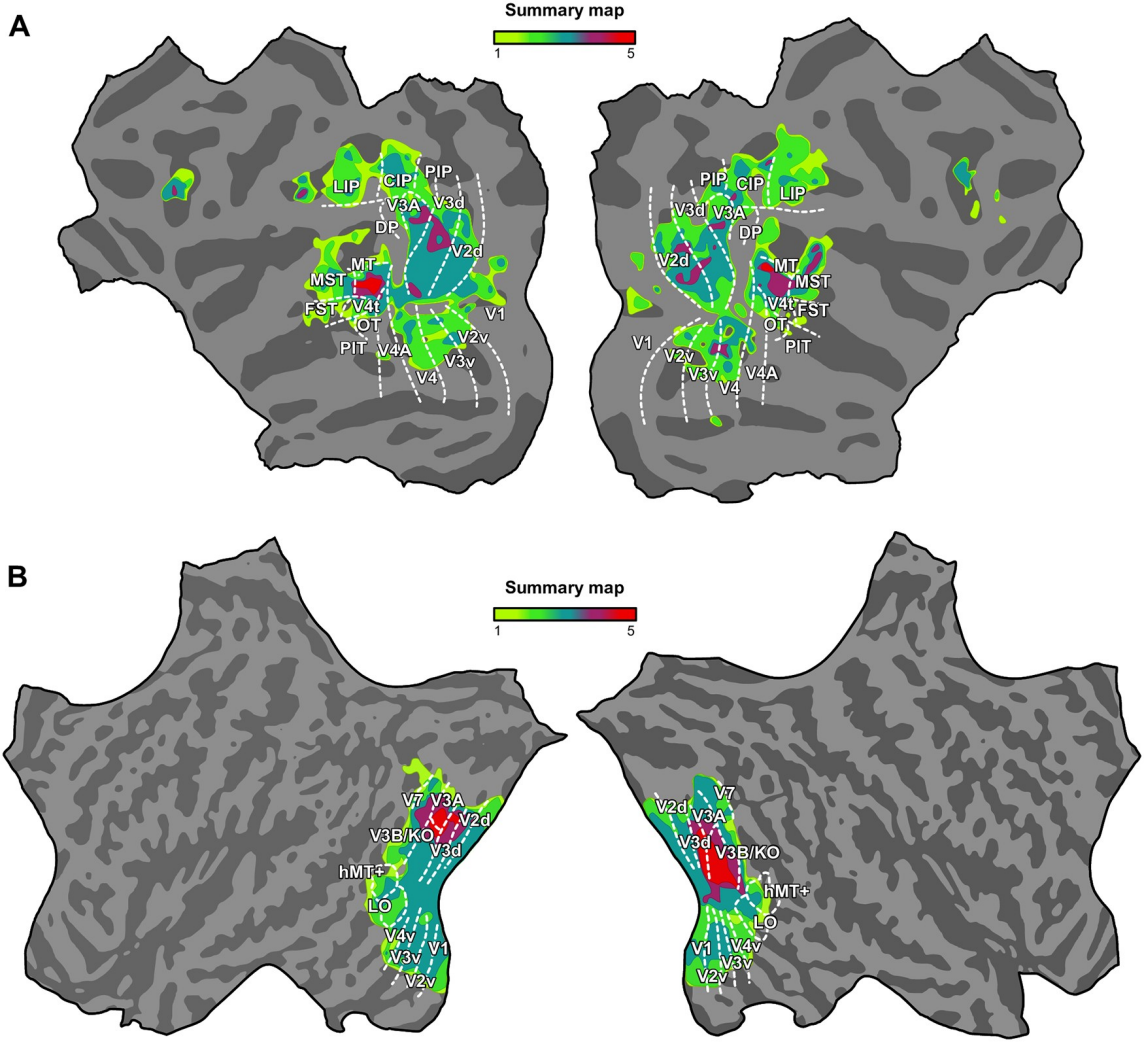
Cue integration summary map in monkey and human. Summary maps highlighting the implication of the different areas in monkeys (A) and humans (B) across all the analyses performed: sensitivity for disparity, motion and congruent stimuli, and integration and transfer indices. Color code indicates each voxel that reached significance (monkey, *P* < 0.01; human, *P* < 0.05) in each of the five tests, ranging from 1 (one test passed) to 5 (five tests passed). The underlying data for the figures can be found at https://doi.org/10.5061/dryad.6pm117m. CIP, caudal intraparietal area; DP, dorsal prelunate area; FST, fundus of the superior temporal sulcus area; LIP, lateral intraparietal area; MST, medial superior temporal sulcus area; MT, middle temporal area; OT, occipitotemporal area; PIP, posterior intraparietal area; PIT, posterior inferotemporal area.

To highlight the implication of areas across the five tests performed (selectivity for disparity, motion and congruent stimuli, and integration and transfer indices), we computed a probabilistic summary map. This color coded map summarizes each voxel that reached significance in each of the five tests (Fig 7). Taken together, these results suggest that monkey area MT and human V3B/KO are the prime candidate regions for fusing motion and disparity cues in the monkey and human brains, respectively.

### Depth discrimination task

**Fig 8:**
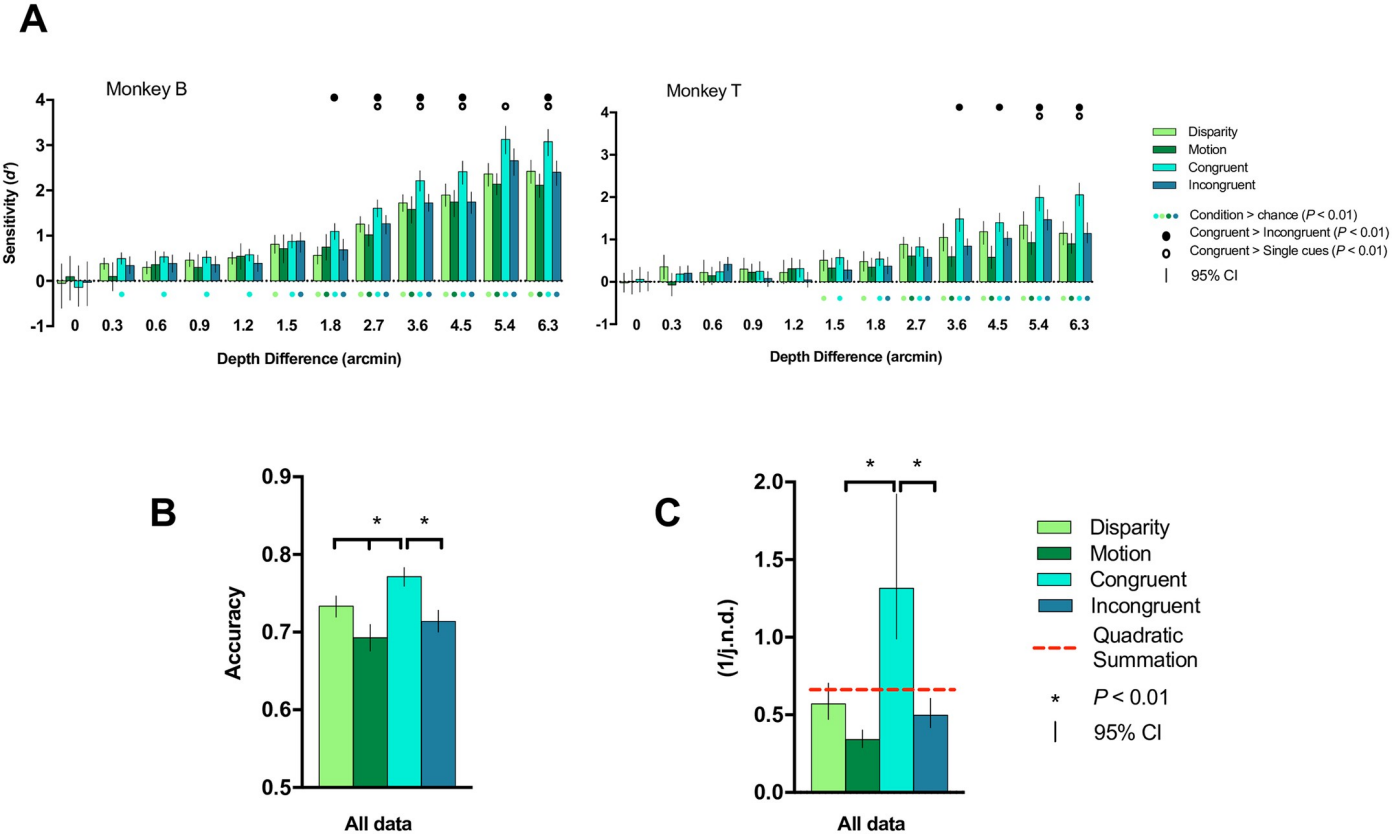
Depth discrimination task. We assessed whether monkeys were able to discriminate different depth levels using the four stimulus conditions of the main experiment. (A) We show sensitivities for depth differences between two sequentially presented planes for monkeys B and T. Both monkeys were able to discriminate between depths for all conditions when the reference and target planes differed by more than 1.8 arcmin in depth. Particularly, monkey B performed excellently and was able to classify between congruent stimuli even for the finest depth difference used (0.3 arcmin). In general, when depths were discriminable, monkeys showed highest sensitivity to the congruent stimulus and lower sensitivity for motion than disparity. Discrimination for the incongruent condition was comparable to that of the single cues. (B) Overall discrimination accuracy across depth levels and monkeys. (C) Sensitivity calculated based on j.n.d. thresholds. As in humans, monkeys were most sensitive when disparity and motion concurrently signalled depth differences, and they were least sensitive for relative motion–related differences. Error bars show bootstrapped 95% confidence intervals; significance was set to *P* < 0.01. The underlying data for the figures can be found at https://doi.org/10.5061/dryad.6pm117m. j.n.d., just noticeable difference.

To assess whether monkeys perceived the depth stimuli in a similar manner as humans (Ban and colleagues, 2012), we performed a depth discrimination task for each of the four conditions in two rhesus macaques. Two planes with different depths were sequentially presented, and monkeys had to indicate whether the second stimulus (target depth) was nearer or farther compared with the first stimulus (reference depth) by making saccadic movements to one of two dots on the left and right sides of the screen. Differences between the reference and target depth stimuli ranged between 0 and 6.3 arcmin. Both monkeys were able to discriminate between the two depth planes (*P* < 0.01) for all conditions at depth differences higher than 1.8 arcmin (Fig 8A). Remarkably, one of the monkeys was able to classify between stimuli even for the smallest tested depth difference (0.3 arcmin) when the congruent condition was presented. In general, when depths were discriminable, monkeys showed higher sensitivity (*d’*) to the congruent stimulus compared with the other conditions. Sensitivity to the relative motion condition was slightly lower than for disparity in both monkeys, while discrimination for the incongruent condition was comparable to that of the single cues. Overall, when pooling the performances across all depth levels and subjects, classification accuracies were above chance for all stimuli and significantly higher for the congruent condition than for the three other conditions (Fig 8B). Moreover, we calculated sensitivity based on just noticeable difference (j.n.d.) thresholds [10]. As in humans, we found that monkeys were most sensitive when disparity and motion concurrently signalled depth differences, and they were least sensitive for relative motion–related differences (Fig 8C). We used sensitivities to single cue conditions to calculate their quadratic summation. In line with fusion and the previous human results [10,11], performance for congruent cues exceeded both the quadratic summation and that of the incongruent cues. In summary, these results suggest similar depth perception across conditions between monkeys and humans, with increased performance for the congruent condition and significantly lower discrimination for relative motion–defined depths compared with depths defined by disparity.

### Eye movement analyses

We analyzed horizontal eye movements from our subjects to assess possible differences between the stimulus conditions. First, we observed that the distribution of eye positions during each condition (averaged across blocks) was centered within a 0.25° window surrounding the fixation point (0°) for all eight conditions (S11A Fig). Second, for each condition, we computed the mean eye position within each presentation block. No significant differences were observed across conditions in eye positions (Kruskal–Wallis test, *P* = 0.1315) (S11B Fig). Hence, differences in fixation are an unlikely explanation of our findings. These results are in line with previous human data using identical stimuli [10], also showing that eye positions were not different across conditions, and unlikely to explain the observed differences in fMRI signals.

## Discussion

Interacting with objects is contingent on a reliable estimation of the 3D structure of the world around us. The primate brain exploits a range of sources of sensory information for that purpose. Amongst others, cues such as binocular disparity and relative motion have shown to be important for humans and monkeys to perceive depth [2,20,21]. Little is known, however, about how these two sources—and others—are combined in the visual cortex to support reliable judgments of depth.

We considered two scenarios under which depth might be processed within the primate visual cortex: a fusion mechanism, in which cues are combined to estimate depth by reducing variance, and independent processing of separate cues, in which different configurations of multivariate distributions (bivariate in our study) can be discriminated using the optimal decision boundary. Our findings in monkey area MT, together with those discovered in human area V3B/KO [10], suggest that a fusion mechanism may take place in dorsal visual areas of the primate brain.

Here, we assessed fMRI responses in monkey visual cortex to near and far depth planes defined by binocular disparity, relative motion, and their combination (both congruent and incongruent). We found that much of the visual cortex is implicated in depth cue processing, although performance varied across regions and conditions (Fig 2). Integration and transfer index maps (Fig 6) highlighted the importance of dorsal visual areas (V2d, V3d, V3A), CIP, and area MT and its satellites in the integration of binocular cues.

We then performed a ROI-based MVPA on fMRI signals and assessed results using three criteria. First, we tested for regions where performance for congruent cue stimuli exceeded the quadratic summation of the marginal cues’ accuracies and hence surpassed the performance expected if depth representations from disparity and motion were collocated but independent. Second, to test whether this improvement was specific to the congruent condition, we contrasted performance when the two cues signalled either the same or the opposite depth. Third, we assessed whether regions supported transfer of depth information between cues.

Our results revealed that MT in the monkey was the only region that met all three criteria. We note that, besides MT, the MST area performed above zero for the ROI-based integration index test, but it did not meet the other two criteria for cue integration. The searchlight maps for the integration index also pointed to CIP and confined subcompartments of V2d and V3d. It is reasonable to think that the increased decoding performance in these areas for the congruent cues is a consequence of collocated representations of disparity and motion information. Consequently, a lack of sensitivity of our analyses for the single cue stimuli in these regions resulted in low decoding performance. Besides MT, area V3d was the only region that passed the transfer test, but V3d did not pass the other two tests. It may be that our tests were not sensitive enough to reveal conclusive evidence for fusion in this area. Decoding accuracy for the disparity condition was very high (>90%) in V3d, and responses for the congruent condition may have been near the ceiling, limiting the capability to exceed the quadratic summation level (Fig 4).

In summary, these findings suggest that area MT plays a key role in the integration of different depth cues in the monkey cortex. Moreover, other extrastriate visual areas (such as V3d) might be related to intermediate depth representation, thus supporting fusion computations in addition to independent representation of depth cues.

### Depth processing in the primate cortex

Depth representation from individual cues (binocular disparity, motion, texture, shape, shading, etc.) has been widely studied; however, surprisingly little is known about how the primate brain integrates the information from different depth cues. Previous fMRI studies in humans reported sensitivity to binocular disparity at multiple levels of the visual hierarchy, from early visual cortex to parietal and temporal areas [22–29]. Likewise, large portions of human extrastriate visual cortex are activated by 3D structure from motion [30–33]. Nevertheless, this extensive sensitivity for both cues does not necessarily imply depth selectivity: for instance, it might represent low-level image features, local disparities, or speed of movement (although this was controlled for in some experiments [5]).

Depth processing has also been investigated in macaques by measuring fMRI responses to both binocular and monocular cues [5,34–39]. Similar to humans, these studies suggested a widely distributed network for 3D representations, implicating monkey occipital, temporal, and parietal cortices. However, the human intraparietal sulcus (IPS) has shown much stronger sensitivity to motion-defined 3D than the corresponding monkey region, suggesting that some visuospatial processing areas might not be present in the macaque cortex [5,36,40] or that similar functions in both species rely on different regions [15].

Behavioral experiments have been performed to compare cue integration in human and nonhuman primates (macaques). Notably, Schiller and colleagues [21] investigated how disparity, motion parallax, and shading depth cues were perceived and integrated. They showed that both species effectively utilized these cues to perceive depth. Moreover, they found that performance was significantly increased when all three cues were presented together, indicating that these separate cues are integrated at yet unknown sites in the primate brain. In addition, consistent with our behavioral data, previous work reported less effective depth discrimination for motion than for disparity in primates [41]. In line with these findings, results from our depth discrimination task in monkeys using disparity and relative motion, together with those previously obtained in adult and juvenile humans [10,11], confirmed the role of these cues in depth perception and that depth cue integration takes place in the primate brain. Overall, similar behavioral results in both species also indicate that monkeys are an excellent animal model for the study of the underlying neural mechanisms of depth perception.

### MT is likely to contain a mixed population of units that support cue fusion

Neurophysiological evidence in nonhuman primates suggests sensitivity to binocular disparity throughout visual cortex [40,42,43]. Recordings in monkey V2 and MT have shown neurons selective to different disparities and clustered organization according to their disparity preference [9,44]. Furthermore, monkey MT has also been related to depth representation defined by motion [4,6]. A recent study [3] showed that single MT neurons have depth-sign–tuned responses for both binocular disparity and motion cues. Specifically, they observed that 68 cells (approximately 51%) preferred either disparity or motion alone, while the remaining cells showed significant selectivity for both conditions. Among these neurons selective for both cues, 37 cells (approximately 28% of the population) had the same depth-sign preference (congruent cells), and 29 cells (approximately 21% of the population) had opposite depth-sign preference (incongruent cells). Thus, disparity tuning at the single cell level did not always match across cues, supporting the existence of congruent and opposite depth-sign–tuned cells. Moreover, responses of congruent neurons to the congruent combination of binocular disparity and motion cues showed an increased selectivity compared with that from single cues, but no enhancement was found in the incongruent cells (when disparity and motion were presented congruently). In Fig 1C we depicted the two ideal scenarios for fusion and independence; however, in light of previous findings, it is unrealistic to expect such a pure neuronal assembly, but a hybrid arrangement instead. On one hand, if a sizeable neuronal subpopulation contributes to fusion, we should be able to detect a sensitivity increase caused by the reduced variance, (only) when the congruent stimulus is presented. At the same time, in a mixed population, separate portions of neurons respond independently to each of the single cues, and their aggregate neuronal response patterns will also be more discriminable when cues are presented together. Moreover, the increase in discriminability by these independent assemblies should equally affect the incongruent condition, because the improvement corresponds to the quadratic sum of the discriminabilities of the marginal distributions. Critically, the contribution of the fusion mechanism will elicit a significant sensitivity improvement that will exceed the quadratic summation of the single cues for the congruent condition, but not for the incongruent stimulus. The combination of both effects can be seen in our results in monkey MT (S10 Fig, left), where (i) a fusion-tuned subpopulation contributes with a substantial enhancement that exceeds the performance of the incongruent condition and the quadratic summation of the individual cues; and (ii) separate single cue–tuned neural subpopulations contribute with higher responses for the incongruent condition compared with single cues, although not higher than their quadratic summation. Altogether, the findings of Nadler and colleagues [3] are in agreement with our results, because we obtained higher classification performance when the two cues are presented congruently (S10 Fig). To the best of our knowledge, responses of congruent cells to the incongruent condition and responses of incongruent cells to both congruent and incongruent conditions were not tested in Nadler and colleagues [3], nor elsewhere [45]. These findings support MT as a potential candidate for integrating depth cues, although the role of opposite cells is currently not understood. Additional electrophysiological studies, in which both congruent and conflicting combinations of disparity and motion cues are tested, are required to further our understanding of cue integration mechanisms at the single cell level in MT.

At a mechanistic level, it is important to note that contributions made by either “congruent” or “incongruent” neurons, when estimating environmental properties from the combination of signals, are unknown. Recently, however, we proposed a model whereby both congruent and incongruent responses contribute to estimating the most likely depth of the scene (at a population level) [46]. In particular, a population “best guess” estimate is computed by combing the outputs of congruent neurons that drive excitation and incongruent neurons that drive inhibition. This process allows robust perceptual estimates—accounting for improved performance when signals are consistent, and robust reversion to one of the signals under conflict. This model explains why many neurons are incongruent, and how the use of such neurons supports robust perceptual estimations by the brain. Our fMRI logic is based on separating “independence” versus “fusion.” It is not specific to the way in which fusion is implemented, and, as corroborated by the new model, it is likely to involve the contributions of both “congruent” and “incongruent” neurons. A given stimulus will evoke activity within the population of fusion of neurons (some will appear “congruent” and others “incongruent,” while, in reality, all encode information about the likelihood of the viewed stimulus). Thus, there is nothing special about the response that will be evoked by a consistent or inconsistent stimulus in terms of the types of neurons that they will stimulate. However, there is a difference in the statistical evidence in favor of a particular depth interpretation: this will be higher when cues are consistent. The present results invite future studies on the neural implementation of the new model [46] at the single cell level in monkey area MT.

### Human V3B/KO versus macaque MT

Recent tests for cue fusion revealed V3B/KO as the main cortical locus in the human cortex for the integration of depth defined by binocular disparity and motion cues [10,11]. Complementary fMRI studies that used texture [47], shading [48], and gloss [49] supported the capability of human area V3B/KO to integrate qualitatively different depth cues. However, no evidence of integration was found in human MT+. Considering that no conclusive evidence yet exists concerning the existence of a macaque homologue of human V3B/KO, one could argue that cue fusion for motion and disparity in monkey occurs in a region more caudally relative to MT, in a location that corresponds at least topographically with V3B/KO. In this respect, it is also worth mentioning that the parcellation of dorsal extrastriate visual cortex of the monkey is highly complex and that the exact areal definitions are highly disputed. Nevertheless, our results suggest an areal difference across primate species for depth cue integration and a potential homology between human V3B/KO and monkey MT for this specific functionality.

Then, can we claim that human MT+ is not an exact homologue to monkey MT? Of course, it is unreasonable to expect that 100% identical areas can be found in two different species, as ecological pressure must have triggered (slightly) different functional properties in individual species. There is evidence that human MT+ and monkey MT might share a large range of functions. For example, when we previously showed monkeys and humans identical videos and correlated the “free-viewing” fMRI signals from independently identified monkey MT with all signals from all voxels of the human cortex, we found significant correlations not only in human area MT+ but also in dorsal areas of the visual cortex. However, when seeding in human MT+, the correlations we found were surprisingly well confined to area MT in the monkey [15]. These entirely data-driven results suggest that MT shares a number of functional properties across species, but not all. While human MT+ is functionally more closely related to MT than other areas in the monkey cortex, monkey MT might be carrying some functionalities that through evolutionary pressure are distributed across several regions of the human visual cortex (S8 Fig). Thus, according to our results and those of Ban and colleagues [10], human MT+ cannot be considered an exact homologue of monkey MT, at least with regard to depth cue integration. Response patterns across species differed substantially in MT (see S9A Fig). Although responses for disparity, relative motion, and their combination are discriminable in both human MT+ and monkey MT (indicating that there is not a lack of responsivity in MT of both species), human MT+ did not show significant increases for the congruent condition compared with the quadratic summation of the single cues, nor a significant transfer of depth information across cues—which are required to support fusion. Moreover, when comparing the relative performances of MT across species, we observed that monkey MT exceeded human MT+ significantly (S9B Fig). Considering these and previous findings, monkey MT share depth cue fusion processes more with human V3B/KO, compared with human MT. However, this specific correspondence does not rule out other functional correspondences between human and monkey MT.

CIP has also been implicated in processing depth from different cues (disparity and texture) and integration [50,51]. In our study, we observed higher classification accuracy for the congruent condition compared with the quadratic summation of the single cues and the incongruent condition in area CIP; however, results were not significant (Fig 4). In addition, near-far discriminability for the motion-defined depth condition dropped to chance level in area CIP, as well as in the rest of parietal regions except for LIP. Accordingly, human V7, the putative corresponding area of monkey CIP [52], did not show selectivity for depth cues integration [10,47].

### What is the contribution of relative motion and speed?

To depict depth structure from motion, we used a display in which near and far conditions were defined by differences in speed between a target (center) and reference plane (surround). On one hand, the reference plane always moved horizontally (and sinusoidally, from left to right and right to left) with a constant speed. Importantly, the direction of the reference plane movement was unambiguous due to a static reference background surrounding the reference plane (Fig 1). When the centrally presented target plane moved faster than the reference plane, it appeared to protrude towards the viewer (near percept). The opposite effect (far percept) was established when the target plane moved slower compared with the reference. Because there is no ambiguity on the direction of the reference plane movement, the differential movement velocity disambiguated the sign of depth in our stimuli [53,54]. Moreover, the occlusion of dot patterns at the edge between target and reference planes may also contribute to solve depth-sign ambiguity [55,56]. It has been shown that accretion–deletion in the presence of relative motion provides sufficient information to disambiguate depth sign. However, accretion–deletion alone is unable to provide any depth perception [56]. Results from our depth discrimination task in monkeys (Fig 8), together with those previously reported in humans [10], suggest that both human and nonhuman primates are capable to discriminate between near versus far depth positions on the basis of relative motion defined in this study.

As in any fMRI experiment, responses from voxels reflect a complex mixture of neural signals (originating from thousands of neurons), most of them even unknown. It is therefore natural to ask whether the fMRI decoding performance that we measure reflects differences in the perceived depth, as opposed to simpler differences in the speed of motion. In particular, it is well known that MT contains neurons that respond differentially to different speeds of motion [57]. Our data contain a number of lines of evidence suggesting that speed per se is unlikely to be responsible for the results that we report. First, the congruent and incongruent stimulus conditions both contained stimuli that differed in disparity-defined depth and the speed of motion. An explanation for our data premised on motion speed alone would not predict the differential decoding performance we find for congruent versus incongruent stimuli in monkey MT. Specifically, the speed of motion for the congruent condition, near disparity and near motion (NN), is matched to that of the incongruent condition, far disparity and near motion (FN), and the speed in the congruent condition, far disparity and far motion (FF), is matched with that of the incongruent condition, near disparity and far motion (NF). Thus, if discrimination relied on speed, the classification of congruent conditions (NN versus FF) and incongruent conditions (NF versus FN) should be similar. Our results in monkeys (MT) and humans (V3B/KO), however, showed differences in performance between congruent and incongruent conditions. This suggests that there is something particular about the difference in speed, in that it produces an impression of depth compatible with the depth signal provided by the disparity cue. Second, if the classification were a mere consequence of speed differences, we would not have expected to see transfer of decoding performance between the different cue conditions (Fig 5). It might be possible to explain this finding based on an association between speed and disparity preference in the underlying selectivities of individual neurons. However, although MT neurons with strong speed tuning also tend to have strong disparity tuning, there is no evidence for a correlation between the preferred magnitude of disparity and velocity [58]. Thus, there is little evidence to suggest that retinal speed signals are confounded with the disparity sign of the stimuli. Although we are aware that fMRI responses contain a complex mixture of neural signals with different properties, such as speed preferences, we believe that the tests performed in our experiment are sufficiently robust to overcome such difficulty. Together, this suggests that the results we report in monkey MT and human V3B/KO are likely to be due to the impression of depth evoked by different speeds of motion, rather than speed.

### Conclusion

We found that fMRI responses are more discriminable when the two cues signal depth concurrently, and that depth information provided by one cue might be diagnostic of depth indicated by the other. We revealed that monkey area MT shows fMRI signals consistent with a fusion mechanism of independent depth cues. These results may reconcile the human imaging data with previous monkey electrophysiological studies implicating area MT in depth perception based on motion and binocular disparity signals. Our findings, together with those obtained in humans, provide evidence for a fusion mechanism for depth perception in the dorsal stream of primates. The fusion of depth cues, however, appears to be computed in different areas in humans (V3B/KO) and monkeys (MT). Therefore, it is tempting to speculate that human V3B/KO may have been part of the MT cluster in an ancestor of monkeys and humans, which has drifted in a caudo-dorsal direction during human evolution.

## Materials and methods

### Subjects

Four rhesus monkeys (*Macaca mulatta*, two female), 4–6 years old, weighing between 4 and 7 kg, participated in the experiments (two underwent fMRI and the other two performed the behavioral task). Animal care and experimental procedures met the Belgian and European guidelines and were approved by the ethical committee of the KU Leuven Medical School (Protocols P103/2008 and P022/2014). Animals were born in captivity and were pair- or group-housed (two to five animals per group; cage size at least 16–32 m^3^) with cage enrichment (toys, foraging devices), outside views, and natural day-night cycles (throughout the year, supplemented with an artificial 12/12-hour light/dark cycle) at the primate facility of the KU Leuven Medical School. They were daily fed with standard primate chow supplemented with bread, nuts, raisins, prunes, and fruits. The animals received their water supply either during the experiments or in the cages before and after the experiments.

Monkeys had previous experience performing behavioral tasks and were prepared for fMRI sessions. Prior to scanning, monkeys that underwent fMRI were trained daily (2–4 weeks) to perform a passive fixation task while in a sphinx position with their head rigidly fixed in a plastic primate chair. For the discrimination task, subjects were trained to answer using saccadic eye movements to left or right targets. Details concerning head-post surgery and training procedures have been previously described [59]. During the experimental period, access to water was restricted, but animals were allowed to drink until fully satiated during the daily training and scanning sessions.

The human data were the same as those presented in Ban and colleagues [10], although additional data analyses were performed on this data set (see below). Because the basic cue-fusion results observed in human V3B/KO described in Ban and colleagues [10] have already been replicated using exactly the same stimuli [11] and with entirely different stimuli [47,48], we deemed it unnecessary to replicate them in the present study. Despite our efforts to perform an identical experiment, there are always unavoidable differences between monkey and human fMRI experiments, which are summarized in S1 Table. One difference is that humans performed a subjective assessment of eye vergence [10], while monkeys received liquid reward to maintain fixation to a fixation point. However, it is rather unlikely that differences in the fixation task greatly impacted the sensory-driven activations. In particular, previous experiments showed that activity patterns can be similar during anesthesia versus awake fixation experiments [60].

### Stimuli

Stereoscopic presentation and display parameters were matched as closely as possible to those used in the previous human study [10] and will be only summarized briefly. For a detailed table listing every difference between the monkey and the human study, see S1 Table.

Stimuli consisted of two planes, the reference plane and the target plane, defined by random patterns of dots (0.15° size, 15 dots/deg^2^ density). The target plane was represented by a square (10 × 10°) located in the center of the screen, superimposed on the rectangular reference plane (18 × 14°). In addition, this random dot region was surrounded by a static grid (43 × 32°) of black and white squares that provided an unambiguous background (permanently present on the screen on top of a gray flat surface). At the center of the screen, a fixation square (0.5° × 0.5°) was presented within a circular mask (1° diameter). The reference plane remained the same across all conditions, with no stereoscopic structure and a constant motion profile. The stereoscopic structure of the dots and the motion of the target plane were altered to depict depth relative to the reference plane. We defined eight stimuli and four different conditions: depth defined (i) by disparity, (ii) by motion, (iii) by congruent disparity and motion, and (iv) by incongruent disparity and motion.

Disparity-defined stimuli were rendered as red-cyan anaglyphs. To create the random dot stereograms (RDSs), we first measured the distance between the centers of the two eyes of our subject to adjust the dot displacements between the left- and right-eye images to the interpupillary distance of each monkey. The luminance of the red dots through the red filter was 11 cd/m^2^ and 0.2 cd/m^2^ through the cyan filter, and the luminance of cyan dots was 10.1 cd/m^2^ through the cyan filter and 0.0 cd/m^2^ through the red filter. For the disparity-alone condition, the target plane was given a horizontal binocular disparity of ±9 arcmin to signal near or far depth relative to the reference plane that presented no binocular disparity structure. Both target and reference planes moved rigidly (same speed and phase) with a sinusoidal horizontal movement of 0.9° amplitude and 1-second period (i.e., in 1 second, the planes covered a displacement of 0.9° from left to right and from right to left).

To depict depth from relative motion alone, the target plane moved horizontally with different amplitudes (1.32° for near and 0.29° for far) relative to the reference plane that kept the same movement amplitude (0.9°). Both planes moved in phase and followed a sinusoidal velocity profile with a 1-second period (Fig 1). The direction of the movement was always obvious due to a static background surrounding the moving reference plane (see above). The relative motion of the target plane with respect to the reference plane gave rise to a pattern of deletion and accretion of the reference (near stimuli) or target (far stimuli) dots, as the two planes translated back and forth across the screen. Because there is no ambiguity about the direction of the reference plane movement due to the static background, the speed difference between planes supports the disambiguation of depth in our stimuli (i.e., when the target moves faster than the reference, it always appears to be protruding toward the viewer) [53].

The near/far speeds of the relative motion stimuli were defined based on the psychophysical experiments [10], during which human observers were asked to judge the impression of depth perceived from relative motion differences of the target and reference planes (the reference plane speed was fixed) and depth perceived from the binocular disparities. We took the psychophysical matching point between the disparity and relative motion stimuli for performing the main fMRI experiments. Even when the eye and/or head movements are restricted, as in the present study and that of Ban and colleagues [10], the depth sign from relative motion was not ambiguous for our stimuli. Our introspection of having viewed these stimuli many times, and the reports of the human observers, indicate that our stimuli were effective in depicting near versus far depth positions on the basis of relative motion.

For stimuli concurrently depicting disparity and motion-defined depth, the target plane had a disparity of ±9 arcmin and a movement amplitude of either 1.32° or 0.29°. In the congruent condition both disparity and motion signalled the same depth (near-near or far-far). For the incongruent case, disparity and motion simultaneously provided opposing information (near-far or far-near).

### Experimental setup and design of the fMRI experiment

Monkeys were placed within the bore of the magnet in sphinx position inside a plastic primate chair using a physical head restraint. Images were projected (Barco 6300 LCD projector) on a translucent screen located at a distance of 57 cm from the monkey. Subjects had to fixate passively on a square presented in the center of the screen. Eye position was monitored at 120 Hz using a pupil-corneal reflection tracking system (Iscan). To encourage monkeys to maintain fixation and remain quiet, liquid reward was delivered through a plastic tube located just inside their mouths. Stimuli were presented to the monkeys through colored filters (red-cyan anaglyph goggles, as in Durand and colleagues [34] and Tsao and colleagues [38]) placed in front of the eyes. Before each scanning session, a contrast agent, MION, was injected into the femoral/saphenous vein (6–11 mg/kg) to improve the contrast-to-noise ratio [59,61].

We presented the stimuli in blocks of 16 seconds. In each block, stimuli were picked randomly from a set of 24 example stimuli (per subject) that differed in the random placement of dots making up the stereogram. Individual stimuli were presented for 1 second, followed by a 1-second fixation period. Three blocks of each stimulus type were randomly presented during an individual run (24 stimulus blocks), and the scan started and ended with a 16-second fixation interval that served as baseline. Each scan lasted 416 seconds, during which 208 volumes were acquired.

### fMRI data acquisition

Monkey data were acquired with a 3T MR Siemens Trio scanner with an AC88-insert head gradient. Functional images were collected using a gradient-echo T2*-weighted echo-planar imaging sequence (repetition time [TR] = 2,000 ms, echo time [TE] = 17 ms, 52 slices, voxel size = 1 mm isotropic, flip angle = 75°). Monkeys were scanned with a custom-built, eight-channel, implanted phased-array receive coil [62] and a saddle-shaped, radial transmit-only surface coil.

To provide an anatomical reference for the functional scans, high-resolution T1-weighted images were acquired for each monkey during a separate session under ketamine-xylazine anesthesia, using a single radial transmit-receive surface coil and a MP-RAGE sequence (TR = 2,200 ms, TE = 4.05 ms, flip angle = 13°, 208 slices, voxel size = 0.4 mm isotropic). During the session, 12–15 whole-brain volumes were obtained and averaged to improve signal-to-noise ratio.

### fMRI preprocessing

Functional volumes were reconstructed online using Siemens GRAPPA image reconstruction. We preprocessed the fMRI data using custom Matlab (MathWorks) scripts and the SPM5 software package (Wellcome Department of Cognitive Neurology, London, UK; http://www.fil.ion.ucl.ac.uk/spm/). We only analyzed runs in which monkeys performed more than 97% of fixation within a 2 × 2 deg. fixation window. Temporal preprocessing was applied to correct for linear trends and spin-excitation history effects caused by head motion. Spatial preprocessing consisted of motion correction and rigid coregistration to the individual anatomical template. To compensate for echo-planar distortions and intersession variance, functional images were matched to the anatomy, applying nonlinear warping in JIP (www.nitrc.org/projects/jip [63]). No spatial smoothing was performed on the data for the analysis.

For each monkey, we used retinotopic mapping procedures to define the ROIs in the occipital cortex, IT, and posterior parietal cortex. Retinotopic organization in occipital visual areas and IT cortex (V1, V2, V3v, V3d, V4, V4A, V4t, OT, MT, MST, FST, and PIT) of both subjects was found to be highly consistent with previous reports [64]. We used individual retinotopic maps along with previous parcellation schemes [65] to delineate V3A, DP area, and posterior parietal cortex areas (PIP, CIP, and LIP). The anatomical labels were drawn on the inflated cortical surface of each monkey using FreeSurfer (http://surfer.nmr.mgh.harvard.edu) and then projected into the volume space. To ensure that distinct ROIs were not overlapping, we performed an automatic correction using customized Matlab code.

Preprocessing of the fMRI responses and ROI definitions in human are described in Ban and colleagues [10]

### ROI-based multivoxel pattern analysis

We first performed a *t* test to contrast the response to all stimulus conditions versus the fixation baseline across all the runs. Within each ROI, we selected gray matter voxels from both hemispheres and sorted them by their *t* statistic. We then selected the top 150 voxels from each ROI. For smaller cortical areas with less than 150 voxels, we selected all the voxels with a *t* value > 0 [25].

We normalized (*z*-score) each voxel’s time course separately for each experimental run to minimize baseline differences across runs. The data vectors for the multivariate analysis were generated by shifting the fMRI time series by two volumes, to account for the hemodynamic response delay. We then averaged all data points within each block to obtain the voxel patterns. To control for the possibility that classification accuracy was due to a univariate baseline difference, we normalized each pattern vector by subtracting the mean voxel amplitude.

We used a linear SVM (LibSVM) to classify between fMRI response patterns evoked by near versus far stimulus presentations for each condition separately and performed an *n*-fold leave-one-run-out cross-validation, in which data from all runs but one were used as training patterns (24 patterns per run, 6 per condition) and data from the remaining run were used as test patterns. Then, the mean accuracy across cross-validations was used.

To quantify differences in SVM prediction accuracies between combined-cue conditions and the minimum bound prediction, we calculated an fMRI integration index (*ϕ*):

$$\phi =\frac{d^{\prime }{}_{D+M}}{\sqrt{d^{\prime }{{}_{D}}^{2}+d^{\prime }{{}_{M}}^{2}}}-1$$ (1)

where *d*′_*D*+*M*_ is the classifier’s performance in the congruent condition, and *d*′_*D*_ and *d*′_*M*_ are performances for single cue conditions in *d*-prime units, calculated with the following formula:

d′=2×erfinv(2p-1) (2)

where *erfinv* is the inverse error function and *p* is the proportion of correct predictions.

To conduct the transfer test analysis, we first used a recursive feature elimination (RFE) method [66] to select the voxels in each ROI with the highest discriminative power. Then, we used a standard SVM to compute within- and between-cue prediction accuracies. To assess the relationship between transfer classification performance and the mean performance for single cues, we calculated a transfer index (T):

$$T=\frac{2d^{\prime }{}_{T}}{d^{\prime }{}_{D}+d^{\prime }{}_{M}}$$ (3)

where *d*′_*T*_ is the transfer classification performance, and *d*′_*D*_ and *d*′_*M*_ are the performances for single cue conditions. Statistical significance of the results was evaluated using bootstrapped resampling with 10,000 samples.

### Searchlight analysis

We performed a searchlight classification analysis [67] in volume space for each monkey by selecting spherical volumes of cortical voxels within a 3-mm radius, moving voxel-wise through the entire cortical volume. Prior to the analysis, a mask was applied to exclude voxels outside the cortex. We ran three SVM classification analyses (disparity, motion, and congruent and incongruent conditions) discriminating between near-far patterns. We then calculated the fMRI integration index at each voxel location (Eq 1). To assess transfer classification accuracies, we conducted between-cue classification analysis. Finally, we computed group maps for each condition and projected the result onto a representative cortical surface (inherently causing some smoothing). This analysis confirmed that all the relevant voxels in the classification analyses were captured by the ROIs previously defined.

In addition to the ROI-based analysis from Ban and colleagues, 2012 [10], we ran the searchlight (8-mm radius) analysis in humans following the same procedures as described for the monkey. We then computed groups maps and projected the results onto a flattened cortical surface of the human brain.

### Depth discrimination task experiment

As in the fMRI experiments, random dot patterns depicting depth from binocular disparity, relative motion, and the combination of both (congruent and incongruent) were presented to two monkeys. Because the monkeys of the fMRI experiment were not available anymore, two different animals (Monkey B and Monkey T), were trained on this task. Monkeys were placed in sphinx position in front of an LCD screen (57-cm distance) and viewed the stimuli through colored filters (red-cyan). In each trial, two stimuli with a slight depth difference between them were sequentially shown, and subjects indicated which of the two planes was farther by making a saccade to one of two targets (1° dots at 10 deg eccentricity) located on the left and right sides of the screen. The first stimulus (1-second duration, reference depth) systematically showed the same depth as the far stimuli during the fMRI experiment (9 arcmin for disparity and/or 0.29° movement amplitude), whereas the second plane (1 second, target depth) was presented at different levels of depth compared with the reference plane (9 ± 0–6.3 arcmin for disparity and 0.29 ± 0–0.18° for motion). For the case of the incongruent condition, disparity far and motion near were depicted, as in the previous human study [10,11]. Each condition was presented in alternate runs, which consisted of 360 trials (15 trials per 12 levels for near or far depths) in which different depth levels of the same condition were randomly presented. A total of 140 runs were acquired.

### Eye movement analysis

Eye positions of one eye were monitored in both monkeys (Iscan, 120 Hz) while they performed the passive fixation task during fMRI sessions. To assess possible differences in eye positions between conditions, we analyzed horizontal eye movements in detail—which are most relevant for depth-defining stimuli. First, for each block (16 seconds), we measured eye position and calculated the average eye position for each stimulus across trials. The mean eye traces were used to compute the distribution of the eye position for each condition. Second, the mean eye position within each presentation block (across the 16 seconds of trial duration) was computed. Then, fixation per condition and 95% confidence intervals were calculated. A Kruskal–Wallis test was used to calculate significant differences between conditions.

## Supporting information

S1 Fig Individual maps for monkey N. Flat maps showing the left and right cortex in monkey N. The borders between areas are delineated by the white dotted lines. Sulci/gyri are coded in dark/light gray. Superimposed on the maps are the results of the classification performances obtained for depths defined by (A) disparity, (B) relative motion, and (C) the congruent combination of disparity and motion. The color code represents the *t* value of the classification accuracies obtained for each condition. Maps (D) and (E) show the results of the integration and transfer tests based on the searchlight analyses. Color code represents the *P* values obtained from the bootstrap distribution of the integration and transfer indices. (F) Integration summary map. Color code indicates each voxel that reached significance in each of the five tests, ranging from 1 (one test passed) to 5 (five tests passed).
(TIFF)

S2 Fig Individual maps for monkey D. Flat maps showing the left and right visual ROIs in monkey D. Same conventions as in S1 Fig ROI, region of interest.
(TIFF)

S3 Fig Searchlight map for the incongruent condition in monkeys. Flat map showing the cortex of the left and right hemisphere of the monkey. Results of a searchlight classifier analysis that moved iteratively throughout the entire volume of cortex, discriminating between near and far depth positions for the incongruent stimulus (group data, *N* = 2). The color code represents the *t* value of the classification accuracies. The underlying data for the figures can be found at https://doi.org/10.5061/dryad.6pm117m.
(TIFF)

S4 Fig Searchlight map for the incongruent condition in humans. Flat maps showing the left and right human cortex. Same conventions as in Fig 3. The color code represents the *t* value (*N* = 11) of the classification accuracies for the incongruent stimulus. Data are from Ban and colleagues, 2012 [10]. The underlying data for the figures can be found at https://doi.org/10.5061/dryad.6pm117m.
(TIFF)

S5 Fig Integration index in humans. Results for the quadratic summation test shown as an integration index. A value of zero indicates the minimum bound for fusion (the prediction based on quadratic summation). Data are presented as notched distribution plots. The center of the “bowtie” represents the median, the greenish area depicts 68% confidence values, and the upper and lower error bars 95% confidence intervals. *P < 0.05 Bonferroni corrected. Data from Ban and colleagues, 2012 [10].
(TIFF)

S6 Fig Transfer test in humans. Transfer index across regions. A value of 100% would indicate that prediction accuracies were equivalent for within- and between-cue testing. Distribution plots show the median; cyan area and error bars represent the 68% and 95% confidence intervals, respectively. Purple dotted horizontal lines depict a bootstrapped chance baseline based on the upper 95th percentile for transfer obtained with randomly permuted data. *P < 0.05 Bonferroni corrected. Data from Ban and colleagues, 2012 [10]. The underlying data for the figures can be found at https://doi.org/10.5061/dryad.6pm117m.
(TIFF)

S7 Fig fMRI decoding data from monkey MT and results from simulations. We explored the composition of the neuronal population, comparing our simulation results to our empirical data. To evaluate how a population mixture might affect decoding results, we used simulations to vary systematically the composition of the neuronal population, following exactly the same procedures as our previous study (see Fig 6 and Methods section in Ban and colleagues, 2012 [10]). (A) Simulation results show decoding performance of a simulated population of voxels for different compositions of neuronal populations. (B) Real fMRI decoding data from monkey MT. (C) The χ^2^ statistic was used to identify the closest fit between empirical and simulated data from a range of population mixtures. According to the simulations, around 35% neurons were found to be tuned as fusion neurons in MT. Error bars, SEM. The underlying data for the figures can be found at https://doi.org/10.5061/dryad.6pm117m. fMRI, functional MRI; MT, middle temporal area.
(TIFF)

S8 Fig Intra- and interspecies activity correlation maps for MT. In a previous study, we showed monkeys and humans identical videos and correlated the “free-viewing” fMRI signals from independently identified monkey MT with all signals from all voxels of the human cortex, and vice versa. When seeding in monkey MT, we found significant correlations not only in human area MT+, but also in dorsal areas of the visual cortex. However, when seeding in human MT+, the correlations we found were surprisingly well confined to area MT in the monkey. These results suggest that MT shares a number of functional properties across species, but not all. While human MT+ is functionally more closely related to MT than other areas in the monkey cortex, monkey MT might be carrying multiple functionalities that are distributed across several regions of the human visual cortex. fMRI, functional MRI; MT, middle temporal area. *Figure was adapted from Mantini and colleagues*, *2012*, *Supplementary Figure 8* [15].
(TIFF)

S9 Fig Comparison of response patterns in monkey MT and human MT. (A) Response patterns in MT differed substantially across species. Here, we show the prediction accuracy for near versus far classification across conditions in monkey area MT (mMT) and human MT+ (hMT). Responses for disparity, relative motion, and their combination are discriminable in both hMT [10] and mMT, indicating that we have enough sensitivity in MT of both species. While cross-cue classification was significant in mMT, discriminability between depths was not significant in hMT, suggesting no transfer of depth information across cues (disparity and motion) in this area in humans. The horizontal line at 0.5 corresponds to chance performance. Error bars, SEM. (B) To assess the difference across response patterns, we compared the relative performance of mMT and hMT+ under three different conditions. Note that we are not comparing absolute but relative activity levels. First, we show the increase of sensitivity for the congruent condition relative to the quadratic summation of the single cues (integration) in both areas. Results in MT across species were significantly different (*P* < 0.01; Bayes factor [BF] in favor of the hypothesis of a difference between mMT and hMT = 16.75). Second, we compared the performance of the congruent condition with the incongruent condition between species. mMT exceeded hMT substantially (*P* < 0.01; BF = 3.06). Third, we compared the cross-cue transfer of depth information between cues (disparity and motion) in both species. While performance was comparable to the permuted chance baseline for hMT (indicating no transfer of depth information), cross-cue transfer accuracy in monkey MT was significantly higher (*P* < 0.01; BF > 10^3^). Error bars, SEM. Statistical significance of the results was evaluated using bootstrapped resampling with 10,000 samples. BF analysis was based on Dienes 2008 (“Understanding Psychology as a Science: An Introduction to Scientific and Statistical Inference.” Palgrave-Macmillan). The underlying data for the figures can be found at https://doi.org/10.5061/dryad.6pm117m. BF, Bayes factor; hMT, human MT+; mMT, monkey area MT; MT, middle temporal area.
(TIFF)

S10 Fig Performance of the incongruent condition in monkey MT and human V3B/KO. Performance of the incongruent condition compared with the congruent condition and the quadratic summation of the single cues. We observed differences in the performance of the incongruent condition relative to the single cues (that might be explained by differences in the composition of the neural population in the ROI); however, the crucial point is whether the sensitivity for the incongruent condition exceeds the quadratic summation of the single cues. Our statistical tests showed that sensitivity for the incongruent condition was not significantly greater (n.s.g.) than the quadratic summation in monkey MT nor in human V3B/KO. Error bars, SEM; *P < 0.01. Statistical significance of the results was evaluated using bootstrapped resampling with 10,000 samples. The underlying data for the figures can be found at https://doi.org/10.5061/dryad.6pm117m. MT, middle temporal area; n.s.g., not significantly greater; ROI, region of interest; V3B/KO, area V3B, kinetic occipital area.
(TIFF)

S11 Fig Eye movement analysis. We assessed possible differences in eye position between conditions. (A) We show the distribution of the eye positions during stimulus presentation (16 seconds) for each condition averaged across trials. Eye positions were centered within a 0.25° window surrounding the fixation point (0°) for all eight conditions. (B) We show eye positions for each condition. No significant differences were observed across conditions (Kruskal–Wallis test, *P* = 0.1315). Hence, differences in eye position are an unlikely explanation of our findings. Error bars show 95% confidence intervals; significance was set to *P* < 0.01. The underlying data for the figures can be found at https://doi.org/10.5061/dryad.6pm117m.
(TIFF)

S12 Fig Classification performance across areas. Classification accuracies for near versus far discrimination in different ROIs and for different conditions. Error bars show 95% confidence intervals. The underlying data for the figures can be found at https://doi.org/10.5061/dryad.6pm117m. ROI, region of interest.
(TIFF)

S1 Table Differences between human and monkey experiment design. Differences regarding the task and fMRI acquisition between human and monkey experiments. fMRI, functional MRI.
(DOCX)

## Abbreviations

2D: two-dimensional
3D: three-dimensional
AIP: anterior intraparietal area
CIP: caudal intraparietal area
DP: dorsal prelunate area
fMRI: functional MRI
FST: fundus of the superior temporal sulcus area
IPS: intraparietal sulcus
j.n.d.: just noticeable difference
LIP: lateral intraparietal area
LO: lateral occipital
MION: monocrystalline iron oxide nanoparticle
MST: medial superior temporal sulcus area
MT: middle temporal area
OT: occipitotemporal area
PIP: posterior intraparietal area
PIT: posterior inferotemporal area
RDS: random dot stereogram
RFE: recursive feature elimination
ROI: region of interest
SVM: support vector machine
TE: echo time
TR: repetition time
V1: primary visual cortex
V2: secondary visual cortex
V2d: dorsal secondary visual area
V2v: ventral secondary visual area
V3A: visual area 3A
V3B/KO: area V3B, kinetic occipital area
V3d: dorsal visual area 3
V3v: ventral visual areas 3
V4: visual area 4
V4A: visual area 4A
V4t: transitional visual area 4
