# Supplementary figures and images for: Areal differences in depth cue integration between monkey and human
Source: PLoS Biol. 2019 Mar 29;17(3):e2006405. doi: 10.1371/journal.pbio.2006405 (PMC6457573; doi:10.1371/journal.pbio.2006405)

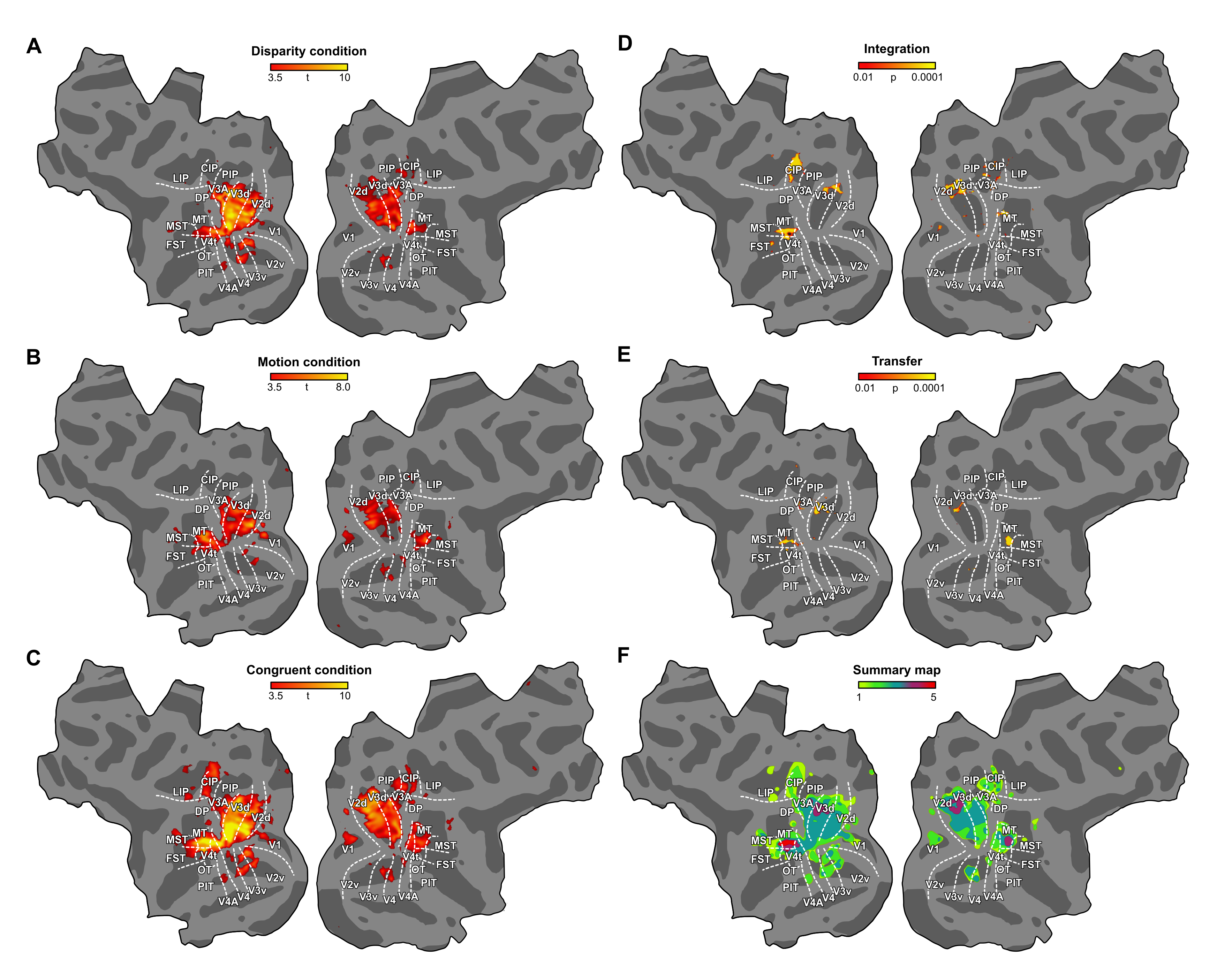

Supplement: S1 Fig — Flat maps showing the left and right cortex in monkey N. The borders between areas are delineated by the white dotted lines. Sulci/gyri are coded in dark/light gray. Superimposed on the maps are the results of the classification performances obtained for depths defined by (A) disparity, (B) relative motion, and (C) the congruent combination of disparity and motion. The color code represents the t value of the classification accuracies obtained for each condition. Maps (D) and (E) show the results of the integration and transfer tests based on the searchlight analyses. Color code represents the P values obtained from the bootstrap distribution of the integration and transfer indices. (F) Integration summary map. Color code indicates each voxel that reached significance in each of the five tests, ranging from 1 (one test passed) to 5 (five tests passed). (TIFF) [file pbio.2006405.s001.tiff]

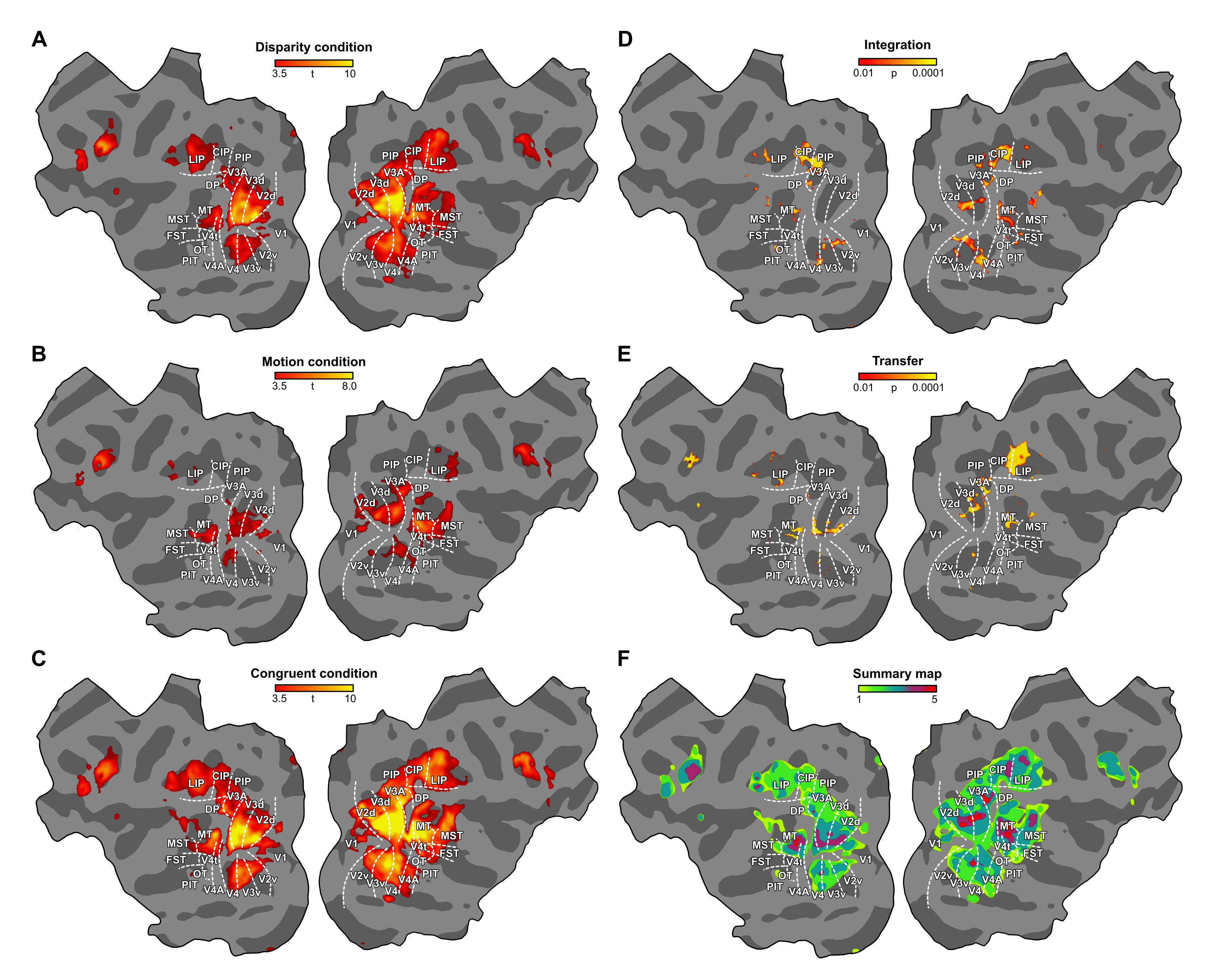

Supplement: S2 Fig — Flat maps showing the left and right visual ROIs in monkey D. Same conventions as in S1 Fig ROI, region of interest. (TIFF) [file pbio.2006405.s002.tiff]

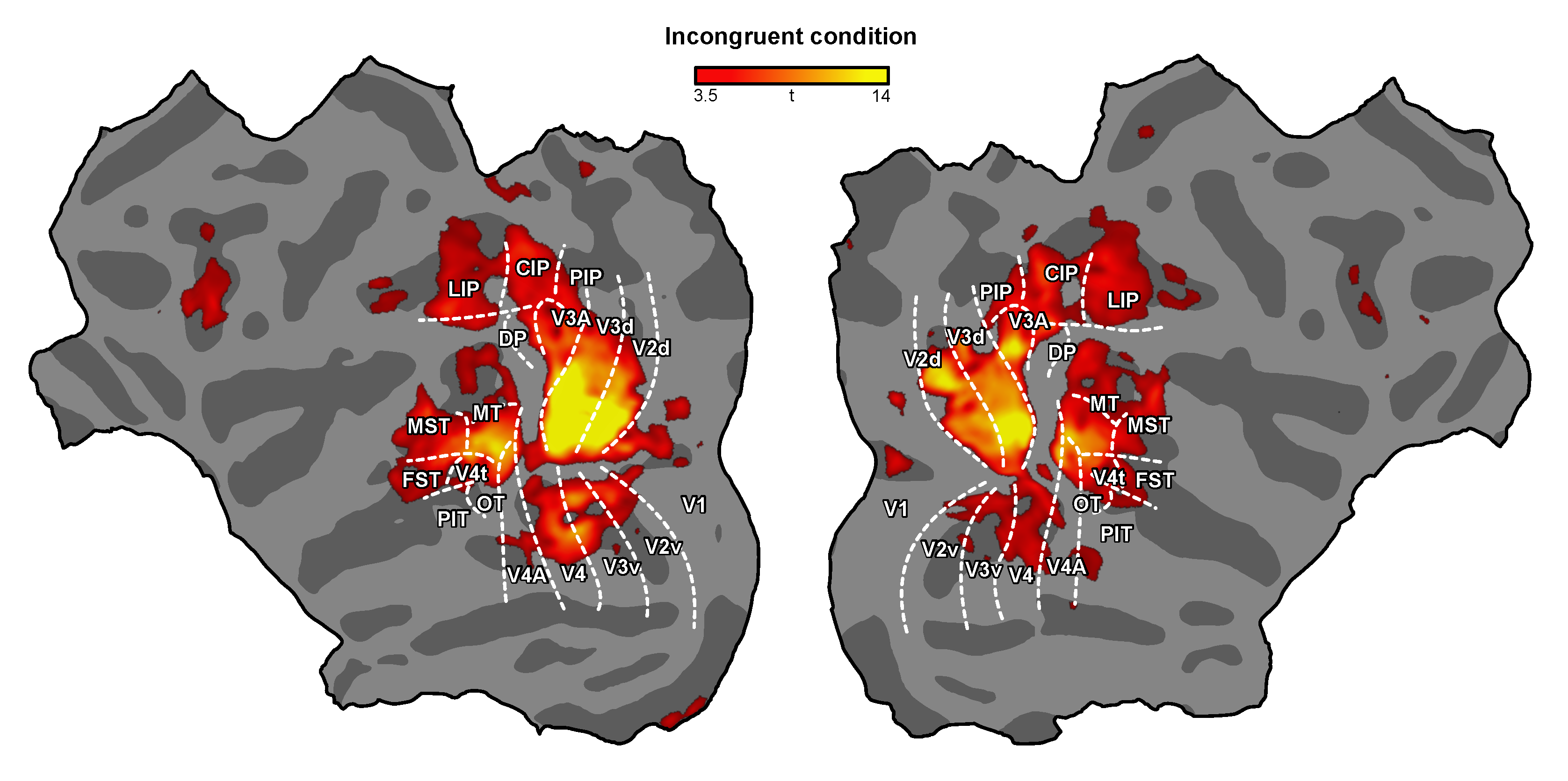

Supplement: S3 Fig — Flat map showing the cortex of the left and right hemisphere of the monkey. Results of a searchlight classifier analysis that moved iteratively throughout the entire volume of cortex, discriminating between near and far depth positions for the incongruent stimulus (group data, N = 2). The color code represents the t value of the classification accuracies. The underlying data for the figures can be found at https://doi.org/10.5061/dryad.6pm117m. (TIFF) [file pbio.2006405.s003.tiff]

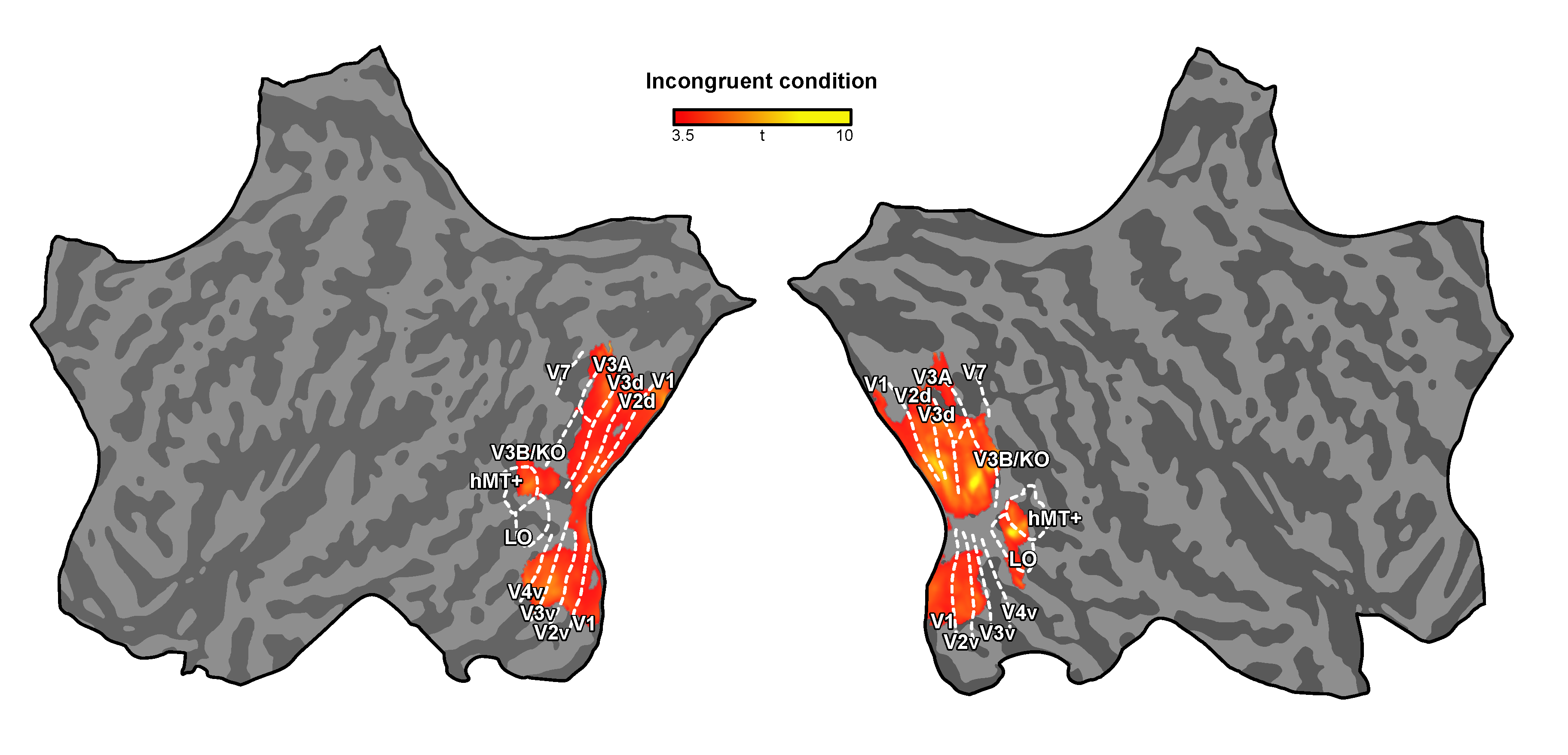

Supplement: S4 Fig — Flat maps showing the left and right human cortex. Same conventions as in Fig 3. The color code represents the t value (N = 11) of the classification accuracies for the incongruent stimulus. Data are from Ban and colleagues, 2012 [10]. The underlying data for the figures can be found at https://doi.org/10.5061/dryad.6pm117m. (TIFF) [file pbio.2006405.s004.tiff]

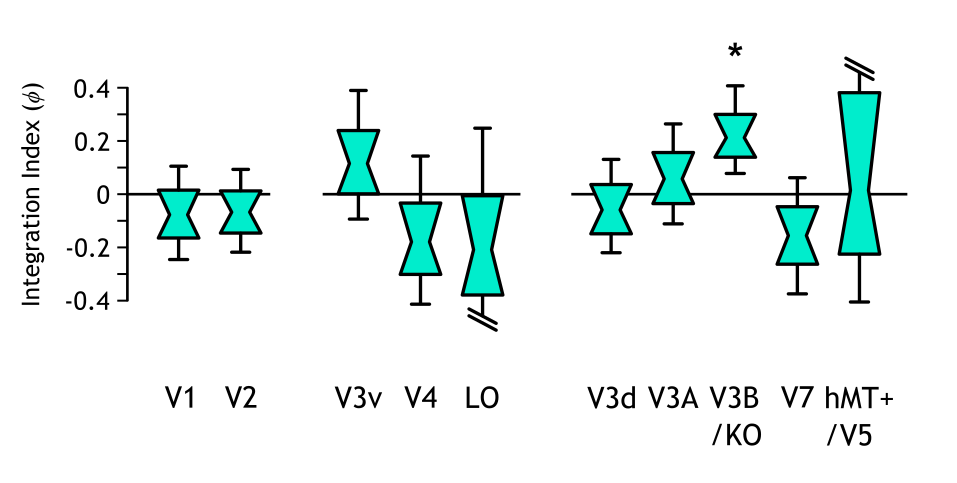

Supplement: S5 Fig — Results for the quadratic summation test shown as an integration index. A value of zero indicates the minimum bound for fusion (the prediction based on quadratic summation). Data are presented as notched distribution plots. The center of the “bowtie” represents the median, the greenish area depicts 68% confidence values, and the upper and lower error bars 95% confidence intervals. *P < 0.05 Bonferroni corrected. Data from Ban and colleagues, 2012 [10]. (TIFF) [file pbio.2006405.s005.tiff]

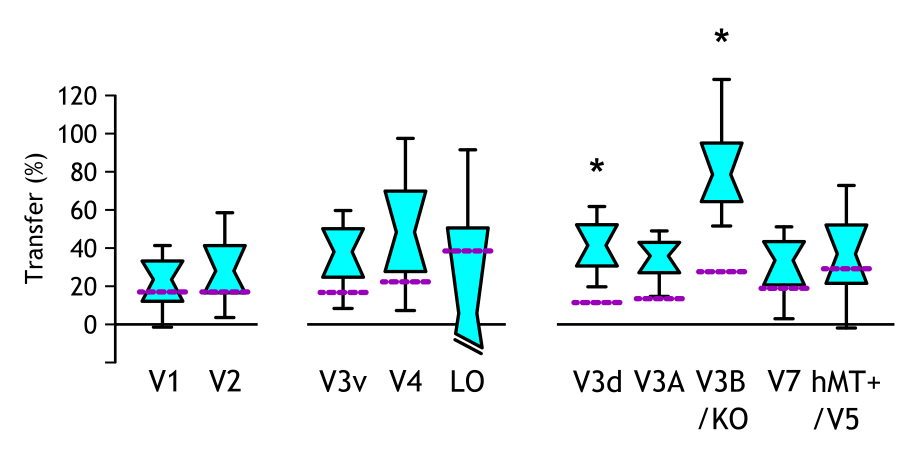

Supplement: S6 Fig — Transfer index across regions. A value of 100% would indicate that prediction accuracies were equivalent for within- and between-cue testing. Distribution plots show the median; cyan area and error bars represent the 68% and 95% confidence intervals, respectively. Purple dotted horizontal lines depict a bootstrapped chance baseline based on the upper 95th percentile for transfer obtained with randomly permuted data. *P < 0.05 Bonferroni corrected. Data from Ban and colleagues, 2012 [10]. The underlying data for the figures can be found at https://doi.org/10.5061/dryad.6pm117m. (TIFF) [file pbio.2006405.s006.tiff]

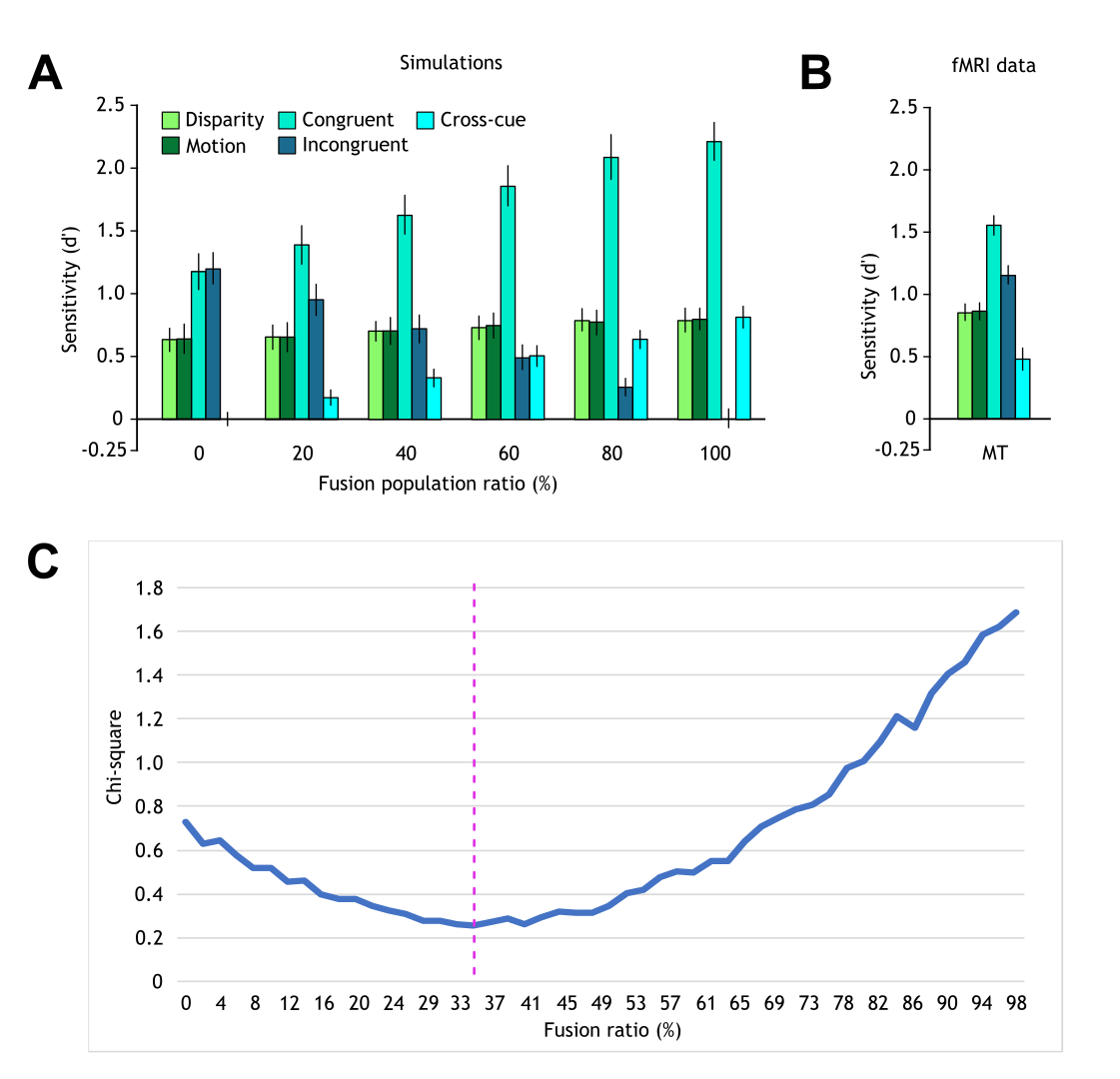

Supplement: S7 Fig — We explored the composition of the neuronal population, comparing our simulation results to our empirical data. To evaluate how a population mixture might affect decoding results, we used simulations to vary systematically the composition of the neuronal population, following exactly the same procedures as our previous study (see Fig 6 and Methods section in Ban and colleagues, 2012 [10]). (A) Simulation results show decoding performance of a simulated population of voxels for different compositions of neuronal populations. (B) Real fMRI decoding data from monkey MT. (C) The χ2 statistic was used to identify the closest fit between empirical and simulated data from a range of population mixtures. According to the simulations, around 35% neurons were found to be tuned as fusion neurons in MT. Error bars, SEM. The underlying data for the figures can be found at https://doi.org/10.5061/dryad.6pm117m. fMRI, functional MRI; MT, middle temporal area. (TIFF) [file pbio.2006405.s007.tiff]

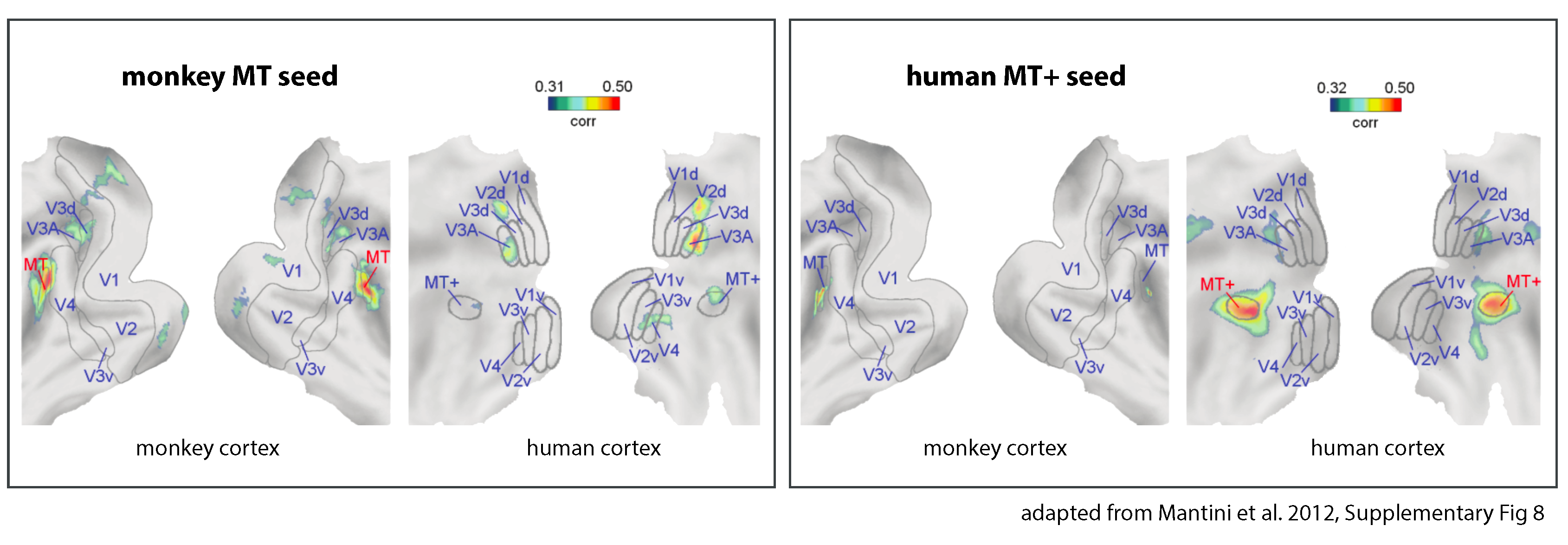

Supplement: S8 Fig — In a previous study, we showed monkeys and humans identical videos and correlated the “free-viewing” fMRI signals from independently identified monkey MT with all signals from all voxels of the human cortex, and vice versa. When seeding in monkey MT, we found significant correlations not only in human area MT+, but also in dorsal areas of the visual cortex. However, when seeding in human MT+, the correlations we found were surprisingly well confined to area MT in the monkey. These results suggest that MT shares a number of functional properties across species, but not all. While human MT+ is functionally more closely related to MT than other areas in the monkey cortex, monkey MT might be carrying multiple functionalities that are distributed across several regions of the human visual cortex. fMRI, functional MRI; MT, middle temporal area. Figure was adapted from Mantini and colleagues, 2012, Supplementary Figure 8 [15]. (TIFF) [file pbio.2006405.s008.tiff]

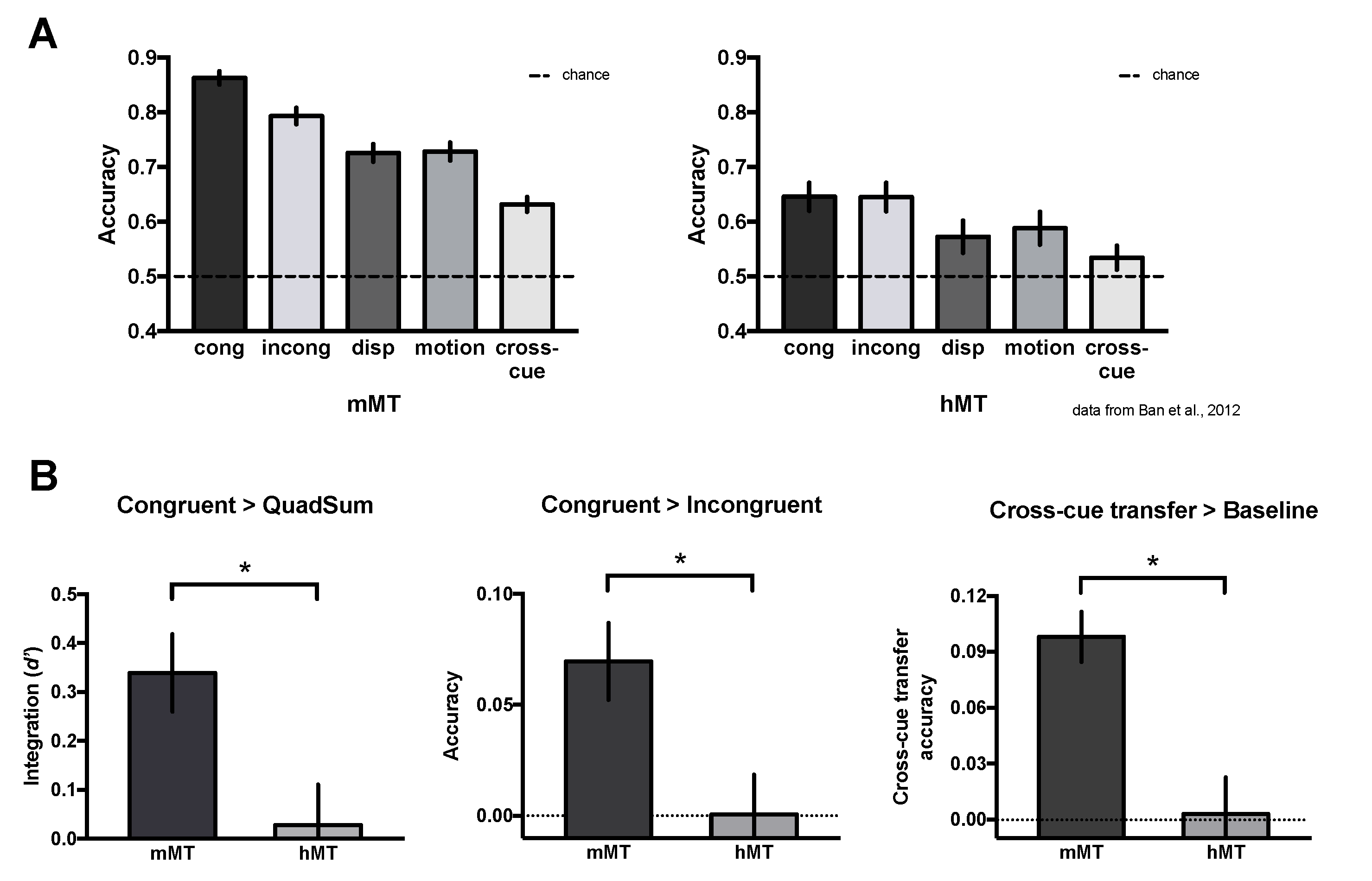

Supplement: S9 Fig — (A) Response patterns in MT differed substantially across species. Here, we show the prediction accuracy for near versus far classification across conditions in monkey area MT (mMT) and human MT+ (hMT). Responses for disparity, relative motion, and their combination are discriminable in both hMT [10] and mMT, indicating that we have enough sensitivity in MT of both species. While cross-cue classification was significant in mMT, discriminability between depths was not significant in hMT, suggesting no transfer of depth information across cues (disparity and motion) in this area in humans. The horizontal line at 0.5 corresponds to chance performance. Error bars, SEM. (B) To assess the difference across response patterns, we compared the relative performance of mMT and hMT+ under three different conditions. Note that we are not comparing absolute but relative activity levels. First, we show the increase of sensitivity for the congruent condition relative to the quadratic summation of the single cues (integration) in both areas. Results in MT across species were significantly different (P < 0.01; Bayes factor [BF] in favor of the hypothesis of a difference between mMT and hMT = 16.75). Second, we compared the performance of the congruent condition with the incongruent condition between species. mMT exceeded hMT substantially (P < 0.01; BF = 3.06). Third, we compared the cross-cue transfer of depth information between cues (disparity and motion) in both species. While performance was comparable to the permuted chance baseline for hMT (indicating no transfer of depth information), cross-cue transfer accuracy in monkey MT was significantly higher (P < 0.01; BF > 103). Error bars, SEM. Statistical significance of the results was evaluated using bootstrapped resampling with 10,000 samples. BF analysis was based on Dienes 2008 (“Understanding Psychology as a Science: An Introduction to Scientific and Statistical Inference.” Palgrave-Macmillan). The underlying data for the fig [file pbio.2006405.s009.tiff]

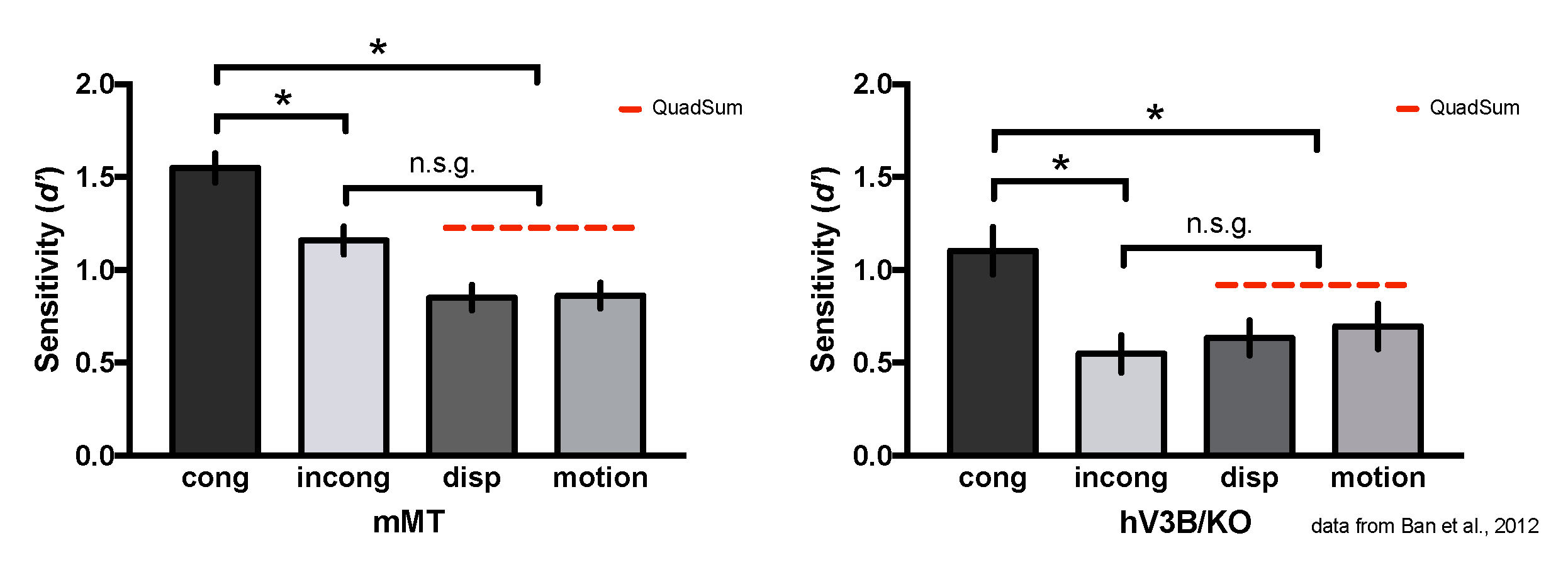

Supplement: S10 Fig — Performance of the incongruent condition compared with the congruent condition and the quadratic summation of the single cues. We observed differences in the performance of the incongruent condition relative to the single cues (that might be explained by differences in the composition of the neural population in the ROI); however, the crucial point is whether the sensitivity for the incongruent condition exceeds the quadratic summation of the single cues. Our statistical tests showed that sensitivity for the incongruent condition was not significantly greater (n.s.g.) than the quadratic summation in monkey MT nor in human V3B/KO. Error bars, SEM; *P < 0.01. Statistical significance of the results was evaluated using bootstrapped resampling with 10,000 samples. The underlying data for the figures can be found at https://doi.org/10.5061/dryad.6pm117m. MT, middle temporal area; n.s.g., not significantly greater; ROI, region of interest; V3B/KO, area V3B, kinetic occipital area. (TIFF) [file pbio.2006405.s010.tiff]

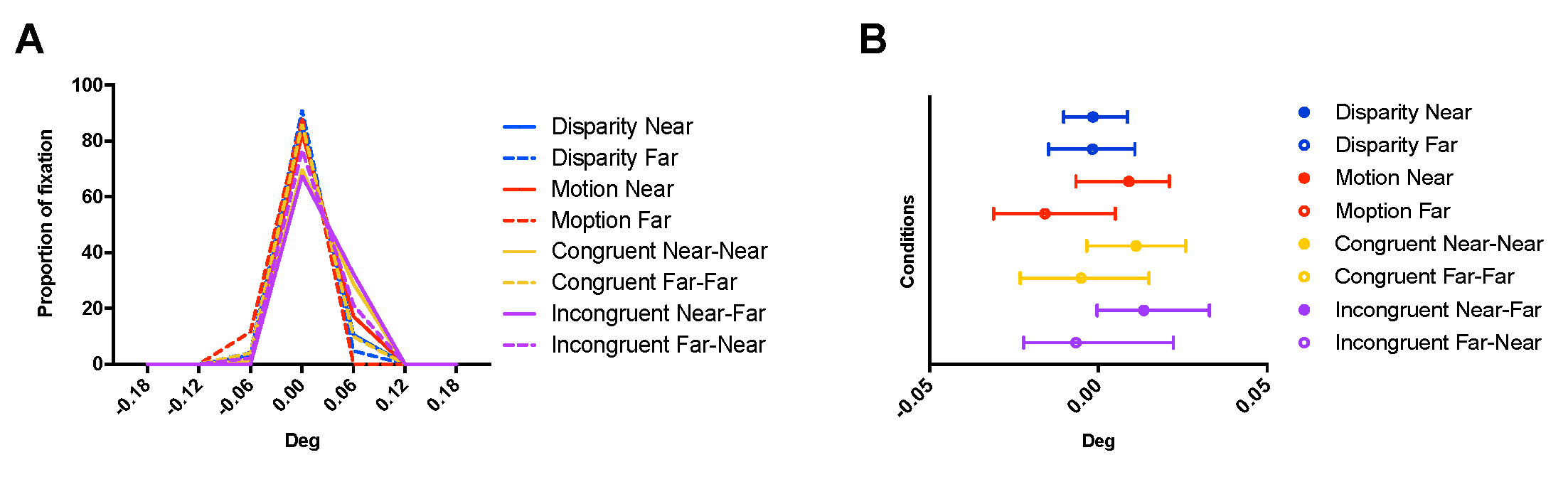

Supplement: S11 Fig — We assessed possible differences in eye position between conditions. (A) We show the distribution of the eye positions during stimulus presentation (16 seconds) for each condition averaged across trials. Eye positions were centered within a 0.25° window surrounding the fixation point (0°) for all eight conditions. (B) We show eye positions for each condition. No significant differences were observed across conditions (Kruskal–Wallis test, P = 0.1315). Hence, differences in eye position are an unlikely explanation of our findings. Error bars show 95% confidence intervals; significance was set to P < 0.01. The underlying data for the figures can be found at https://doi.org/10.5061/dryad.6pm117m. (TIFF) [file pbio.2006405.s011.tiff]

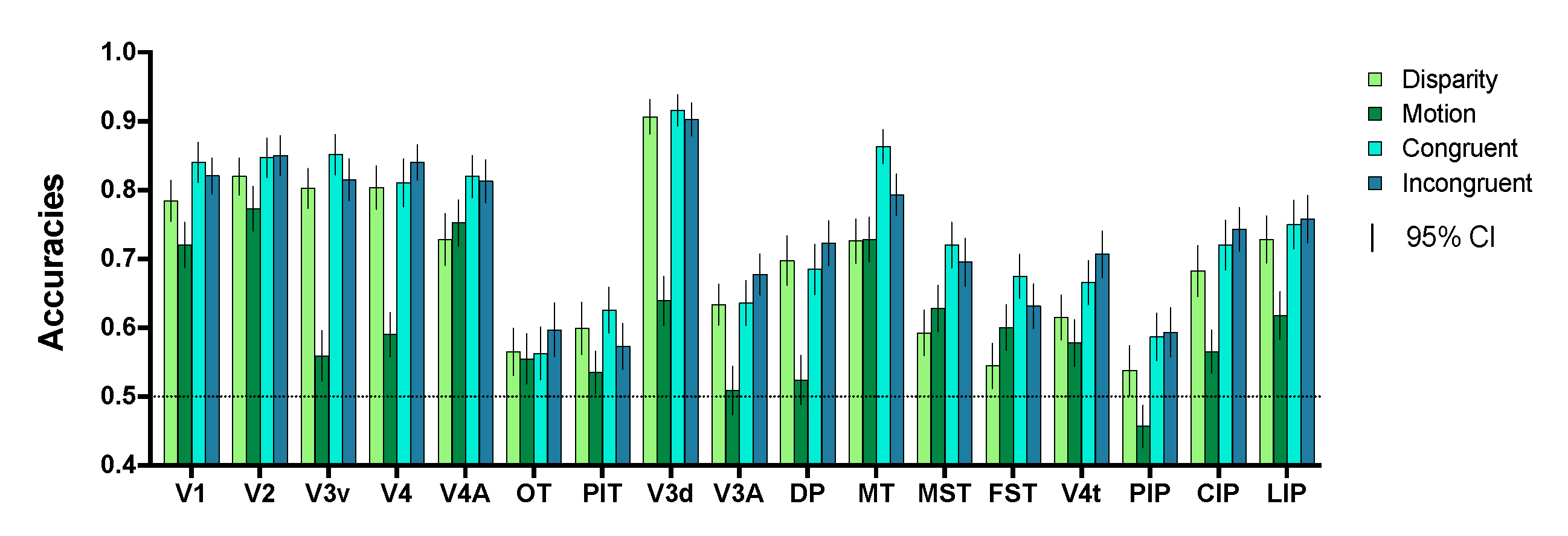

Supplement: S12 Fig — Classification accuracies for near versus far discrimination in different ROIs and for different conditions. Error bars show 95% confidence intervals. The underlying data for the figures can be found at https://doi.org/10.5061/dryad.6pm117m. ROI, region of interest. (TIFF) [file pbio.2006405.s012.tiff]
