# Supplementary material for: Areal differences in depth cue integration between monkey and human
Source: PLoS Biol. 2019 Mar 29;17(3):e2006405. doi: 10.1371/journal.pbio.2006405 (PMC6457573; doi:10.1371/journal.pbio.2006405)
Supplement: S1 Table — Differences regarding the task and fMRI acquisition between human and monkey experiments. fMRI, functional MRI. (DOCX) [file pbio.2006405.s013.docx]

|  | **Monkey experiment** | **Human experiment** |
| --- | --- | --- |
| Task and setup | Subjects performed only fixation to the center of the screen | Subjects fixated to the center of the screen and performed a subjective assessment of eye vergence |
|  | Subjects received liquid reward | Each subject received 20£ |
|  | Subjects sit in sphinx position with their head rigidly fixed viewing the screen through colored filters | Subjects lie on their back viewing the screen through a mirror |
| fMRI acquisition | 3T MR Siemens Trio scanner with an AC88-insert head gradient | 3T Philips Achieva MR scanner |
|  | Contrast agent (MION) | No contrast agent (BOLD) |
|  | Implanted phased-array receive coils (8 channels) | Phased-array Head coil (8 channels) |
|  | Full brain | Posterior part of the brain |
|  | Resolution 1x1x1 mm, 52 slices | Resolution 1.5x1.5x2 mm, 27 or 28 slices |
